# Microbial assimilatory sulfate reduction-mediated H_2_S: an overlooked role in Crohn’s disease development

**DOI:** 10.1186/s40168-024-01873-2

**Published:** 2024-08-16

**Authors:** Wanrong Luo, Min Zhao, Mohammed Dwidar, Yang Gao, Liyuan Xiang, Xueting Wu, Marnix H. Medema, Shu Xu, Xiaozhi Li, Hendrik Schäfer, Minhu Chen, Rui Feng, Yijun Zhu

**Affiliations:** 1 Department of Gastroenterology, the First Affiliated Hospital, Sun Yat-Sen University, No.58 Zhongshan Er Road, Room 1209, Guangzhou, 510080 China; 2 Institute of Precision Medicine, the First Affiliated Hospital, Sun Yat-Sen University, Guangzhou, Guangdong China; 3 Department of Gastroenterology, Shenzhen No.3 People’s Hospital, Shenzhen, Guangdong China; 4 Department of Cardiovascular & Metabolic Sciences, Lerner Research Institute, Cleveland Clinic, Cleveland, OH USA; 5 Center for Microbiome and Human Health, Cleveland Clinic, Cleveland, OH USA; 6 Bioinformatics Group, Wageningen University, Wageningen, The Netherlands; 7 School of Life Sciences, University of Warwick, Coventry, UK; 8 Key Laboratory of Human Microbiome and Chronic Diseases (Sun Yat-Sen University), Ministry of Education, Guangzhou, Guangdong China

**Keywords:** Inflammatory bowel disease, Inorganic sulfate, Sulfopolysaccharide, 3’-Phosphoadenosine-5’-Phosphosulfate PAPS, Adenosine-5’-Phosphosulfate APS

## Abstract

**Background:**

H_2_S imbalances in the intestinal tract trigger Crohn's disease (CD), a chronic inflammatory gastrointestinal disorder characterized by microbiota dysbiosis and barrier dysfunction. However, a comprehensive understanding of H_2_S generation in the gut, and the contributions of both microbiota and host to systemic H_2_S levels in CD, remain to be elucidated. This investigation aimed to enhance comprehension regarding the sulfidogenic potential of both the human host and the gut microbiota.

**Results:**

Our analysis of a treatment-naive CD cohorts' fecal metagenomic and biopsy metatranscriptomic data revealed reduced expression of host endogenous H_2_S generation genes alongside increased abundance of microbial exogenous H_2_S production genes in correlation with CD. While prior studies focused on microbial H_2_S production via dissimilatory sulfite reductases, our metagenomic analysis suggests the assimilatory sulfate reduction (ASR) pathway is a more significant contributor in the human gut, given its high prevalence and abundance. Subsequently, we validated our hypothesis experimentally by generating ASR-deficient *E. coli* mutants *∆cysJ* and *∆cysM* through the deletion of sulfite reductase and L-cysteine synthase genes. This alteration significantly affected bacterial sulfidogenic capacity, colon epithelial cell viability, and colonic mucin sulfation, ultimately leading to colitis in murine model. Further study revealed that gut microbiota degrade sulfopolysaccharides and assimilate sulfate to produce H_2_S via the ASR pathway, highlighting the role of sulfopolysaccharides in colitis and cautioning against their use as food additives.

**Conclusions:**

Our study significantly advances understanding of microbial sulfur metabolism in the human gut, elucidating the complex interplay between diet, gut microbiota, and host sulfur metabolism. We highlight the microbial ASR pathway as an overlooked endogenous H_2_S producer and a potential therapeutic target for managing CD.

Video Abstract

**Supplementary Information:**

The online version contains supplementary material available at 10.1186/s40168-024-01873-2.

## Introduction

Crohn's disease (CD) and ulcerative colitis (UC) are two main forms of Inflammatory Bowel Disease (IBD), characterized by symptoms including diarrhea, rectal bleeding, abdominal pain, fatigue, and weight loss, significantly impacting patients' lives. IBD incidence and prevalence are rising globally, particularly in newly industrialized regions [1, 2]. The growing global burden of this disease underscores the need for preventive and therapeutic measures [2]. Although the precise etiology remains elusive, it is believed to result from dysregulated mucosal immune responses triggered by gut bacteria, especially in individuals with genetic predispositions [3, 4].

Sulfur metabolism and sulfur-containing metabolites play a pivotal role in IBD [5–7]. Hydrogen sulfide (H_2_S) is the sulfur derivative that garners the most attention in the context of colonic health. In the gastrointestinal system, the H_2_S pathway supports epithelial, immune, and enteric nervous system health through various mechanisms, including posttranslational modification of protein cysteine residues, activation of K_ATP_ channels, and serving as an inorganic fuel for colonocytes [8–10]. However, excessive exposure to H_2_S can be detrimental to the host, damaging the intestinal epithelium and leading to chronic inflammation, as well as disrupting the balance between cellular proliferation and apoptosis [11]. An association between elevated H_2_S levels and IBD has long been suspected [12, 13]. Several studies suggest that pharmacological interventions targeting H_2_S may improve outcomes in IBD through mechanisms such as driving regulatory T cell differentiation, stabilizing hypoxia-inducible factor 1-alpha (HIF-1α), promoting biofilm formation, and reducing planktonic bacteria growth [14–16].

However, a comprehensive mechanistic model elucidating the relationship between H_2_S generation and IBD is still lacking. The production and release of H_2_S are regulated by both endogenous and exogenous factors, but the relative contributions of the host and gut microbiota to overall systemic H_2_S levels in humans remain uncertain. Endogenous H_2_S production primarily results from the enzymatic degradation of organic sulfur compounds, particularly cysteine. Key enzymes in this process include cystathionine beta-synthase (CBS), cystathionine gamma-lyase (CTH), 3-mercaptopyruvate sulfur transferase (MPST), and methanethiol oxidase (SELENBP1) [17, 18]. On the other hand, our understanding of microbial-mediated H_2_S generation remains limited. Nevertheless, several studies have shown that bacteria generate H_2_S to mitigate oxidative stress from antibiotics [19], drive cryptic redox chemistry to shape gut metabolism [7], regulate intracellular cysteine levels [20], and influence bacterial virulence via proteome S-sulfhydration [21], highlighting the necessity to clarify the mechanisms of bacterial H_2_S generation.

**Fig. 1:**
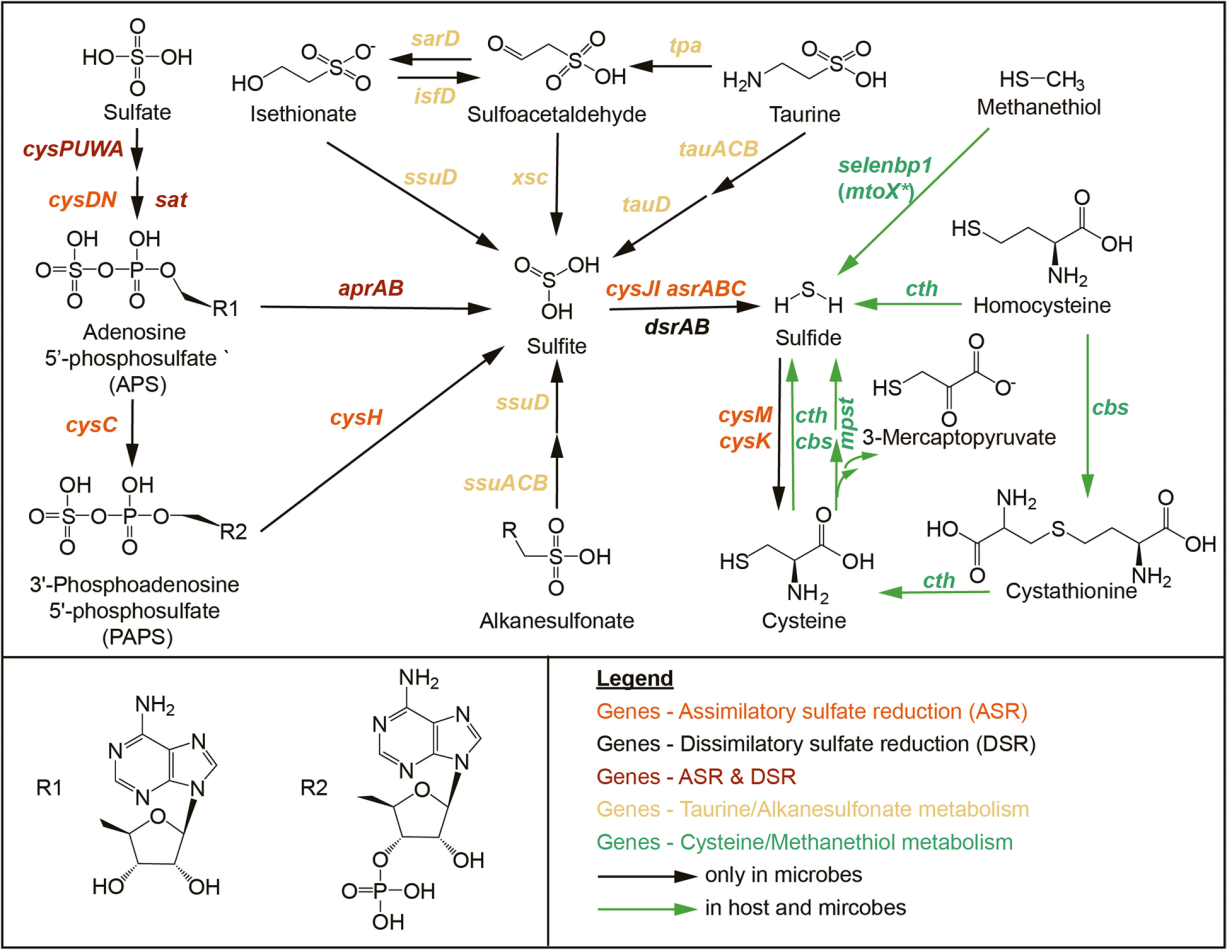
Exogenous microbial sulfur metabolism results in the production of genotoxic H_2_S via metabolism of inorganic sulfate and organic sulfur like cysteine, taurine, isethionate, methanethiol and alkanesulfonate (black and green). Endogenous H_2_S is produced via metabolism of sulfur containing amino acids like cysteine and homocysteine, as well as organic sulfur methanethiol (green). Gene names and KEGG ID are listed in Supplementary dataset 1. * Recent research has demonstrated that sulfane sulfur is the direct product of the bacterial methanethiol oxidase (MtoX) [25]

Bacteria produce H_2_S through the utilization of both organic sulfur compounds like L-cysteine and taurine, as well as inorganic sulfur compounds such as sulfate and sulfite. The two primary pathways for sulfate metabolism are Assimilatory Sulfate Reduction (ASR) [22, 23], involving the reduction of sulfate to H_2_S, which is subsequently incorporated into cysteine and methionine biosynthesis, and Dissimilatory Sulfate Reduction (DSR), a process found in sulfate-reducing bacteria where these microbes produce H_2_S from sulfate without integrating it into L-cysteine [24] (Fig. 1).

Previous investigations into microbial sulfidogenesis found the contributions of gut microbiota to systemic total H_2_S levels varied widely across subjects [26], and have mainly focused on the fermentation of organic sulfur compounds [5, 27] and sulfate-reducing bacteria DSR [28, 29]. Meanwhile, ASR, a common strategy employed by many microbes to fix sulfur and manipulate organosulfur compounds, has been routinely overlooked.

Here, we employed genomic and metagenomic tools to gain a deeper understanding of the colonic sulfidogenic capacity of both the host and gut microbiome in a newly-onset treatment-naïve CD cohort, and observed that CD exhibit reduced endogenous H_2_S production alongside increased gut microbial H_2_S generation, primarily via the ASR pathway. Mechanistically, we genetically manipulated *E. coli* ASR pathway to evaluate the impact on (i) *E. coli*'s sulfidogenic capacity, (ii) colon epithelial cell viability, and (iii) the development of colitis and maintenance of mucus integrity in a mouse model. Our data elucidate a previously unappreciated role of microbial ASR pathway in dietary sulfate metabolism, intestinal sulfur homeostasis and mucus integrity, emphasizing its pivotal role in CD pathogenesis.

## Results

### CD is associated with up-regulation of gut microbial assimilatory sulfate reduction

We conducted a comprehensive investigation into the H_2_S production capabilities of the human gut microbiome, focusing on key genes responsible for sulfide generation from various sources, including organic compounds (such as dietary rich L-cysteine and taurine) and inorganic sulfate (Fig. 1, Supplementary dataset 1). Our analysis was based on stool metagenomic samples from two independent IBD cohorts, FAH-SYSU (treatment naïve IBD cohort enrolled at the First Affiliated Hospital of Sun Yat-sen University) [30] and PRISM (Prospective Registry of IBD study at MGH) [3]. ShortBRED was employed to identify unique sequence markers of related family members and quantifying their relative abundance in metagenomic data with high specificity [31]. It is noteworthy that ShortBRED was not specifically developed for gene cluster identification and quantification. Nevertheless, our search within metagenomic datasets using individual genes revealed a moderate to high degree of consistency among genes from the same cluster (spearman r 0.59–0.94, *p* < 0.001, Supplementary dataset 2), confirming the accuracy of the predictions.

**Fig. 2:**
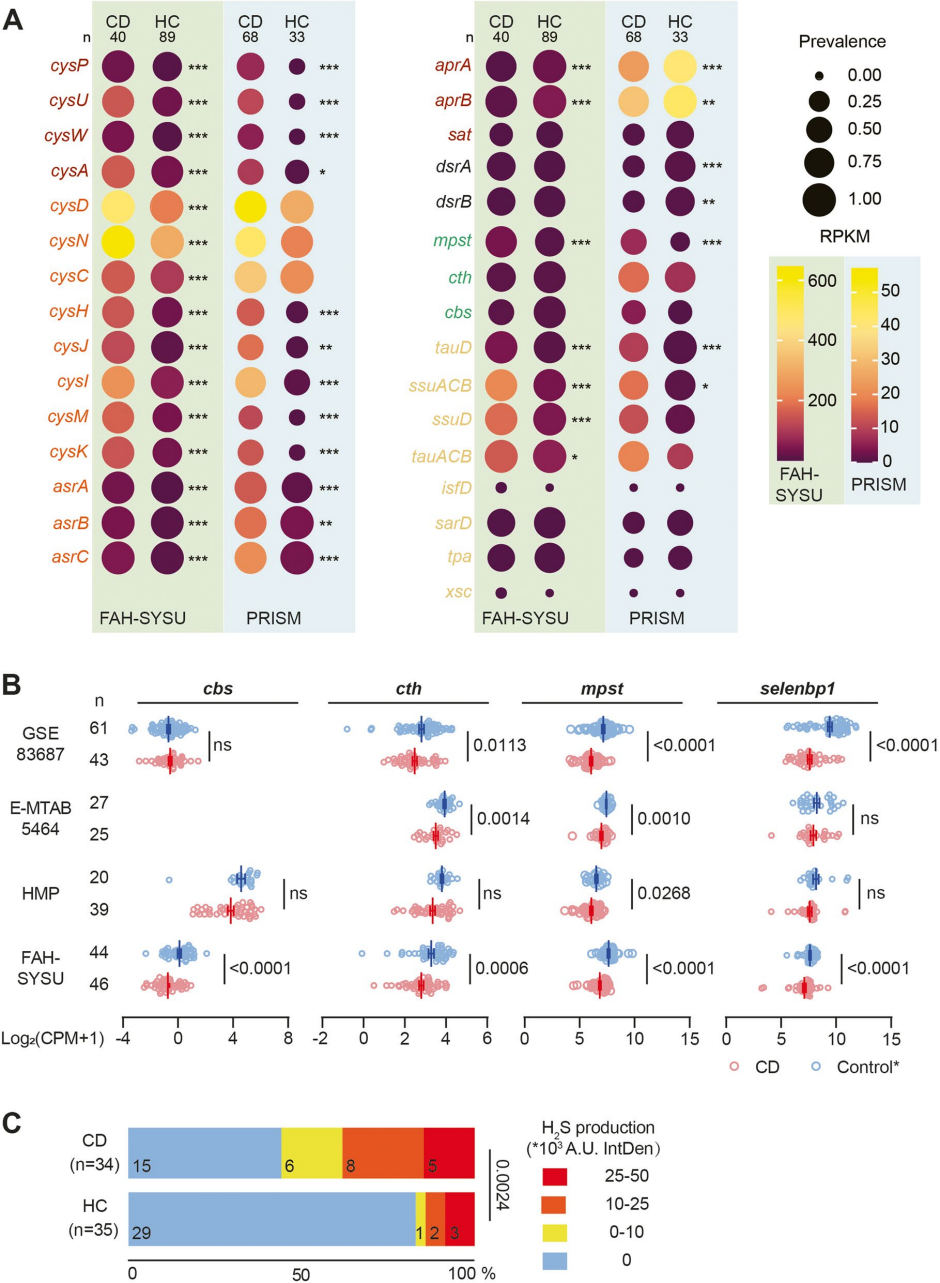
CD is associated with decreased endogenous sulfidogenic gene expression and increased gut microbial exogenous sulfidogenic gene expression. **A** Dot plots comparing selected genes related to microbial sulfide generation from CD versus healthy control subjects (HC) in FAH-SYSU (light green background) cohort and PRISM (light blue background) cohort. The size of each dot indicates the proportion of participants detected in each group of the indicated gene and the color of each dot indicates RPKM with that gene in each group. RPKM, reads per kilobase per million mapped reads. Genes related to ASR, DSR, and organic sulfur metabolism are color-coded according to the scheme in Fig. 1. **B** Analysis of *cbs*, *cth*, *mpst* and *selenbp1* gene expression in CD and non-IBD control subjects’ mucosa in different IBD cohorts. CBS, cystathionine beta-synthase; CTH, cystathionine gamma-lyase; MPST, 3-mercaptopyruvate sulfurtransferase; SELENBP1, methanethiol oxidase. * Various control groups were utilized in different cohorts. FAH-SYSU, non-disease control; HMP, symptomatic non-IBD controls; E-MTAB5464, non-disease control; GSE83687, normal non inflamed bowel away from the tumor from sporadic colon cancer patients. **C** CD patients exhibit increased assimilatory sulfate reduction activity in their fecal microbial community compared to healthy subjects. Significance was determined by nonparametric Mann–Whitney test. *p < 0.05, **p < 0.01, ***p < 0.001

We found that genes associated with ASR, including sulfate adenylyltransferase (*cysDN*) and adenylylsulfate kinase (*cysC*), were highly prevalent in both cohorts. In the FAH-SYSU cohort, these genes were present in 100% of both CD and HC subjects, while in the PRISM cohort, their prevalence ranged from 82 to 100%. These genes were also abundant, with RPKM values of 98.0–646.7 in the FAH-SYSU cohort and 20.6–57.2 in the PRISM cohort for both CD and HC subjects (Fig. 2, Supplementary dataset 3). The PRISM cohort exhibited lower abundance, possibly due to differences in sequencing procedures. In the ASR pathway, organisms use different strategies: 1) Adenosine-5’-phosphosulfate (APS) is phosphorylated into 3’-Phosphoadenosine-5’-phosphosulfate (PAPS) by CysC, which is further reduced into sulfite (SO_3_^2−^) by PAPS reductase (CysH); 2) APS is directly reduced by an APS reductase (AprAB) to generate adenosine monophosphate (AMP) and SO_3_^2−^. Both scenarios generate SO_3_^2−^ which could further be reduced by anaerobic sulfite reductase (AsrABC) [32] or sulfite reductase (CysJI) to form sulfide (S^2−^), which subsequently yield L-cysteine mediated by cysteine synthase A (CysK*)* and cysteine synthase B (CysM) (Fig. 1). We found ASR downstream genes, including *cysH*, *cysJI*, *cysM*, *cysK*, and *asrABC*, were also more abundant in CD subjects (*p* < 0.01), suggesting a significant role for ASR in H_2_S production from SO_4_^2−^ in CD individuals (Fig. 2A, Supplementary dataset 3).

We observed that the prevalence and abundance of *dsrAB* genes (key genes for the DSR but not the ASR pathway) were notably lower compared to *asr*-associated genes in both cohorts. In the PRISM cohort, *dsrAB* genes were detected in approximately 30.9–32.4% of CD subjects, while this percentage increased to 67.6–76.5% in HC subjects. Moreover, their abundance increased from approximately 0.48 to 0.60–0.72 RPKM (*p* < 0.01). However, no significant difference in *dsrAB* genes was observed in the FAH-SYSU cohort (Fig. 2A). Additionally, *aprAB*, responsible for converting APS to SO_3_^2−^ in both DSR and ASR, showed a marked reduction in CD subjects (Fig. 2A). Furthermore, we investigated *cysPUWA*, which encodes a sulfate transporter common to both pathways. These transporter genes were more prevalent and abundant in CD subjects, indicating increased microbial sulfate transport in CD patients.

Organic sulfur metabolism has been reported to be enriched in individuals with IBD and colorectal cancer (CRC) [33, 34]. Therefore, we investigated microbial genes associated with organic sulfur metabolism in our study. We found that the bacterial gene *mpst*, important for converting L-cysteine to H_2_S, exhibited more prevalent and significantly elevated levels in CD subjects compared to HC subjects in both cohorts (*p* < 0.001, Fig. 2A). Additionally, genes related to taurine and alkanesulfonate metabolism, including taurine transporter (*tauABC*), taurine dioxygenase (*tauD*), sulfonate transporter (*ssuACB*), and alkanesulfonate monooxygenase (*ssuD*), were more abundant in CD subjects in FAH-SYSU cohort (*p* < 0.001, Fig. 2A). Microbial methanethiol oxidase (*mtoX*), widely distributed in the biosphere [35], was not detected in human associated bacteria and therefore was excluded in the ShortBRED analysis. In the PRISM cohort, an increasing trend was observed in *tauABC* and *ssuD* among CD subjects, although statistical significance was not attained.

### CD patients demonstrate impaired endogenous H_2_S production

Endogenous H_2_S production arises from the host's utilization of sulfur-containing amino acids (Fig. 1). To shed light on endogenous sulfidogenic activity, we evaluated the expression levels of the host *cbs*, *cth*, *mpst* and *selenbp1* by examining intestinal biopsies obtained from newly diagnosed CD patients (n = 46) and non-disease controls (n = 44) from the FAH-SYSU cohort [36]. Our analysis revealed that all of these 4 genes exhibited significant decreases in inflamed mucosal biopsies from CD subjects (Fig. 2B). A similar trend was observed in three independent IBD cohorts, including the Mount Sinai Hospital cohort (GSE83687) [37], a treatment-naive pediatric IBD cohort (E-MTAB-5464) [38] and the HMP IBD cohort [4] (Fig. 2B), although statistical significance was not achieved in some cases. In the E-MTAB-5464 cohort, transcriptomic data were generated from purified intestinal epithelial cells. CBS raw counts in this cohort were generally less than 10, hence not analysed. This finding strongly suggests a substantial reduction in the endogenous sulfidogenic capacity of CD patients.

To uncover whether CD patients have impaired H_2_S catabolism capacity, we examined the expression levels of key enzymes responsible for host H_2_S catabolism in these cohort datasets, including thiosulfate sulfurtransferase (TST), thiosulfate sulfide:quinone oxidoreductase (SQOR), and persulfide dioxygenase (ETHE1) (Fig. S1A) [13, 39]. Our analysis revealed that *tst* was significantly downregulated, while *sqor* was upregulated in CD subjects in FAH-SYSU cohort. A similar trend was observed in the HPM IBD cohort, although *tst* did not reach statistical significance between CD and non-IBD groups in this cohort. The expression of *ethe1* remained similar in both FAH-SYSU and HMP cohorts, irrespective of CD or control groups (Fig. S1B). Therefore, further research is needed to explore the H_2_S catabolism capacity in CD patients.

### Oxygen-insensitive ASR is functionally more active in fecal microbiota from CD patients

To further substantiate the contribution of the ASR pathway from gut microbiota to H_2_S generation in CD, we conducted an ex vivo fecal culture experiment using thiosulfate (S_2_O_3_^2−2−^) as the sole sulfur source. DSR has been reported in sulfate-reducing bacteria which are strictly anaerobes, whereas ASR has been reported in facultative anaerobes and aerobes [40]. Therefore, we set up the fecal culture aerobically and measured H_2_S production in fecal samples from both healthy individuals and CD patients to test if oxygen-insensitive ASR activity was enhanced in CD’s gut microbiota. We detected H_2_S production in 19 out of 34 (55.9%) CD stool samples, with 13 showing notably high levels (> 10,000 intensity). In contrast, only 6 out of 35 (17.1%) samples from healthy controls exhibited H_2_S production, with 5 demonstrating high levels (Fig. 2C). Thus, oxygen-insensitive ASR is more active in CD patients.

### The bacterial ASR pathway is prevalent in the human microbiome

**Fig. 3:**
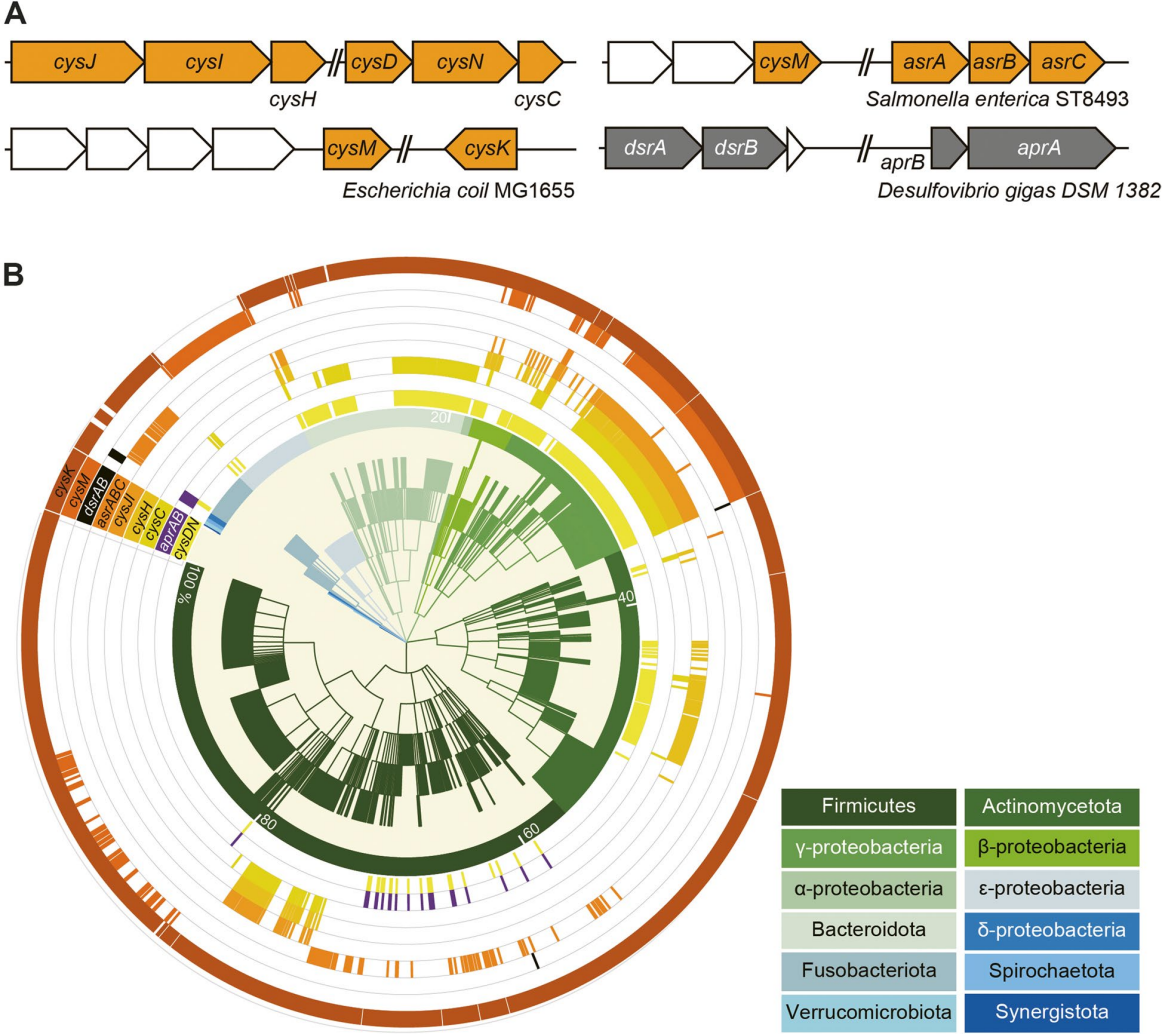
ASR pathway is widely distributed in human microbiota. **A** Arrangement of *asr*- and *dsr*- associated genes and gene clusters in bacteria. Orange ORFs: *asr*-associated genes; gray ORFs: *dsr*-associated genes. Other genes not shown are represented by white ORFs. **B** Phylogenetic distribution of genomes harboing *asr*- and *dsr*-associated genes in 1635 reference genomes of the Human Microbiome Project (HMP). Gene and genome names are listed in Supplementary dataset 4

The *asr* gene cluster in *E. coli* MG1655 [22] and *Salmonella enterica* ST8493 [23], along with the *dsr* gene cluster in *Desulfovibrio gigas* DSM 1382 [24], that have been characterized in previous studies, are shown in Fig. 3A. To comprehensively assess the distribution of *asr*- (*cysDN*, *cysC*, *cysH*, *cysJI*, *cysM, cysK*, *aprAB and asrABC*) and *dsr*-associated genes (*dsrAB*, *aprAB*) among human bacteria, we screened these genes against 1635 Human Microbiome Project (HMP) reference genomes. This extensive analysis revealed that *asr*-associated genes are more widespread than *dsrAB* (Fig. 3B, Supplementary dataset 4). A significant number (88.1%, 1441 out of 1635 reference genomes) of the total reference genomes contain at least one *asr*-associated gene, distributed predominantly in Firmicutes, Actinobacteria, Proteobacteria, and Bacteroidetes, whilst *dsrAB* genes are only found in 0.43% (7 genomes) which are from Firmicutes and γ-Proteobacteria (Fig. 3B, Supplementary dataset 4). The prevalence of *cysDN* (409 genomes) and *cysC* (295 genomes) in Bacteroidetes were higher than the other *asr*-associated genes. On the other hand, *cysJI* (193 strains) and *cysM* (275 strains) were more prevalent in Proteobacteria, including facultative aerobic species like *E. coli*, *Proteus mirabilis*, *Klebsiella oxytoca* (Fig. 3B, Supplementary dataset 4). *asrABC* (117 genomes) was more commonly found in Firmicutes and Fusobacteria. These observations suggest a potential collaborative interplay among microorganisms in the execution of the ASR pathway.

### Construction of *E*. *coli* mutants with impaired assimilatory sulfate reduction (ASR)

**Fig. 4:**
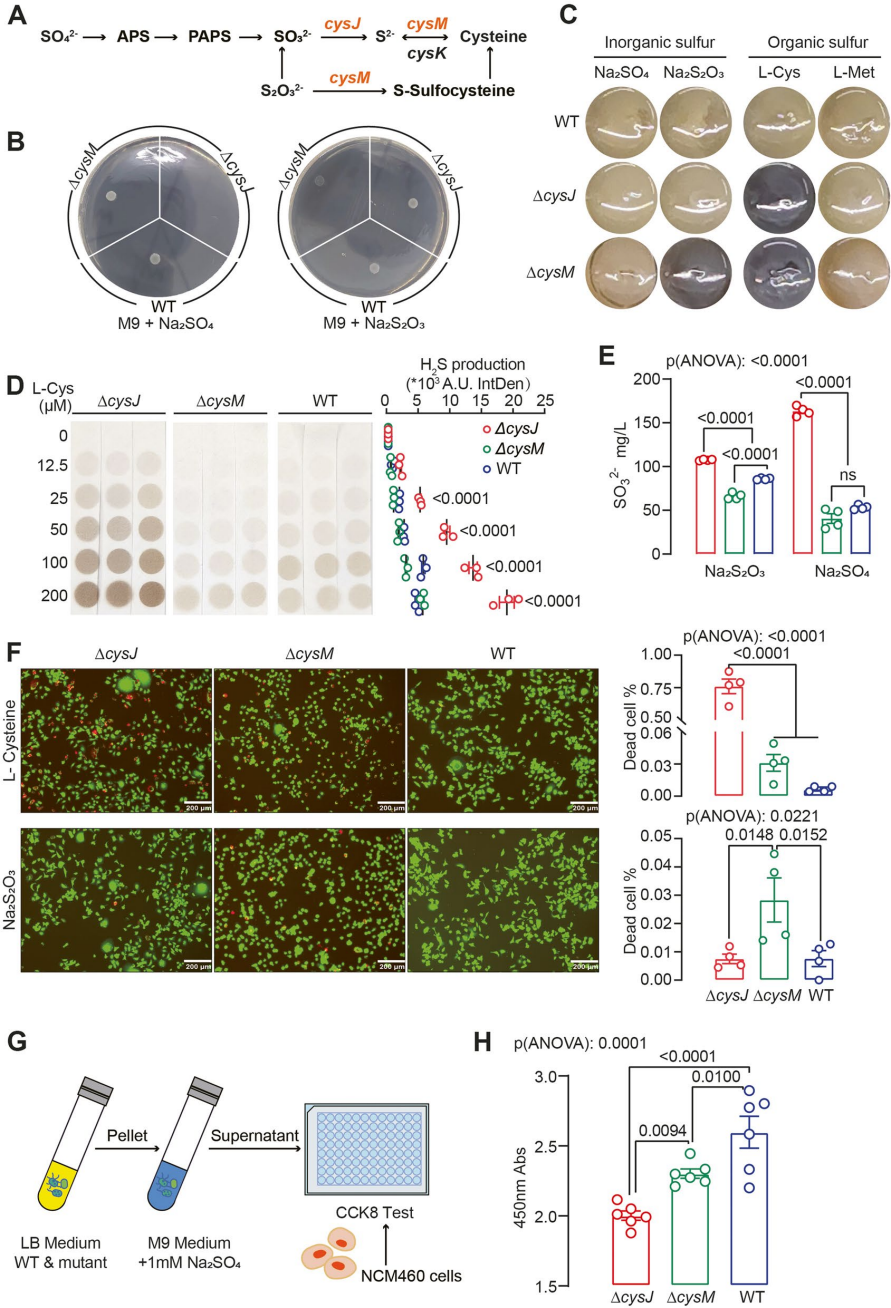
Deletion of *cysJ* and *cysM* alters *E. coli* H_2_S sulfidogenic capacities and modulate cell viability. **A** Scheme of the *E. coli* MG1655 sulfate assimilation reduction pathway. **B** Growth of *E. coli* WT and mutant strains on M9 medium with 1 mM sulfate (left) or sodium thiosulfate (right) as the sole sulfur source. **C** WT and mutant strains of *E. coli* were qualitatively tested for H_2_S in SIM media of different sulfur sources, as evidenced by the formation of black FeS. **D** Relatively quantitative test of H_2_S produced by *E. coli* WT and mutant strains in M9 medium with different concentrations of L-cysteine as a sole sulfur source under aerobic conditions. Significance was measured with two-way ANOVA analysis with Tukey's multiple comparisons. **E** Quantification of sulfite (SO_3_^2−^) produced by *E. coli* WT and mutant strains in M9 medium using 1 mM Na_2_S_2_O_3_ or Na_2_SO_4_ as the sole sulfur source. **F** Representative images and quantification of cell death rates cell death rate of NCM460 cells co-cultured with *E. coli* WT and mutant strains, with either L-cysteine supplementation (upper panel) or sodium thiosulfate supplementation (lower panel), were analyzed using live/dead staining. Living cells are represented in green, while dead cells are shown in red. **G**-**H** A CCK-8 assay was performed using NCM460 cells treated with supernatant from M9 medium containing 1 mM Na_2_SO_4_ that had been pre-inoculated with *E. coli* WT or mutant strains. Mean ± SEM is displayed from at least three independent experiments. Significance was measured with one- and two-way ANOVA analysis with Tukey's multiple comparisons

We focused on the *cysJI*-mediated ASR pathway in this study since metagenomic data indicated that it is more abundant than *asrABC* (Fig. 2A). We used *E. coli* MG1655, a known bacterium with a complete ASR pathway, as the model organism (Fig. 4A). Using homologous recombination, we deleted two crucial ASR pathway genes, *cysJ* and *cysM*. *cysJ* encodes sulfite reductase alpha subunit (*cysI* encodes beta subunit), responsible for the reduction of SO_3_^2−2−^ to S^2−2−^ while *cysM* encodes cysteine synthase B, which converts S^2−^ to L-cysteine. (Fig. 4A). As expected, deleting *cysJ* hindered *E. coli* growth on SO_4_^2−^ as the sole sulfur source (Fig. 4B and Fig. S2A). *E. coli* carries a CysM homologue, CysK, which compensates for CysM in incorporating S^2−^ into L-cysteine (Fig. 4A). Consequently, *E. coli ∆cysM* strains exhibited growth similar to the WT strain when SO_4_^2−^ was the sole sulfur source. Alternatively, CysM can use thiosulfate (S_2_O_3_^2−^) in place of S^2−^ to produce L-cysteine via S-sulfocysteine as the intermediate (Fig. 4A). Therefore, *E. coli* WT and *∆cysJ* mutant grew on S_2_O_3_^2−^ as the sole sulfur source, however *∆cysM* displayed diminished growth rate (Fig. 4B and Fig. S2B).

We assessed *E. coli* WT and mutant strains for their sulfidogenic capabilities using various inorganic and organic sulfur sources. In the modified Sulfur, Indole, Motility (SIM) medium, we observed that deleting *cysJ* increased H_2_S production from L-cysteine, while deleting *cysM* enhanced H_2_S generation from both L-cysteine and SO_3_^2−^ (Fig. 4C). *E. coli* WT also produced H_2_S from L-cysteine, as indicated by slight medium darkening (Fig. 4C). We further cultured *E. coli* WT and mutant strains in M9 medium supplemented with varying concentrations of L-cysteine, and observed that all the strains exhibited a dose-dependent production of H_2_S, with the *∆cysJ* mutant demonstrating greater efficiency in converting L-cysteine to sulfide than *∆cysM* and WT (Fig. 4D). Under anaerobic conditions, *E. coli ∆cysM* actively reduced SO_3_^2−^ to produce H_2_S (Fig. S2C). While the *∆cysJ* mutant consistently showed increased H_2_S production in the presence of L-cysteine under anaerobic conditions, both *E. coli* WT and mutant strains displayed decreased H_2_S production compared to aerobic conditions (Fig. S2D). Additionally, the *∆cysJ* mutant accumulated SO_3_^2−^ in M9 medium when SO_4_^2−^ or S_2_O_3_^2−^ was the sole sulfur source due to the loss of sulfite reductase activity (Fig. 4E). These findings highlight the impact of ASR pathway alterations on both inorganic and organic sulfur metabolism. *E. coli* WT and ASR-deficient mutants displayed distinct morphological characteristics and proteomic profiles (Fig. S3A, B, Supplementary dataset 5), suggesting that the alteration of the ASR pathway has a profound effect on bacterial physiology.

### Bacterial assimilatory sulfate reduction modulates epithelial cell viability

We proceeded to investigate the impact of modifications in the bacterial ASR pathway on the growth of colonic epithelial cells in an in vitro setting. We co-cultured *E. coli* WT and mutant strains with normal human colonic mucosal epithelial cell line NCM460 with either L-cysteine or S_2_O_3_^2−^ as the sole sulfur source. Cell viability assay revealed that in the presence of L-cysteine, the *∆cysJ* mutant led to significantly decreased cell viability, concurrent with increased H_2_S production (Fig. 4D, F). When S_2_O_3_^2−^ served as the exclusive sulfur source, the *∆cysM* mutant induced more pronounced cell death, accompanied by higher H_2_S generation (Fig. 4C, F).

As *∆cysJ* mutant accumulates more SO_3_^2−^ in the medium when SO_4_^2−^ is the sole sulfur source (Fig. 4E), this accumulation of SO_3_^2−^ may potentially leads to cell toxicity [41]. To investigate it further, we collected the supernatants from cultures of *E. coli* WT and mutant strains grown in M9 medium supplemented with Na_2_SO_4_ and used them to treat NCM460 cells (Fig. 4G). As indicated by the cell proliferation assay, the *∆cysJ* mutant exhibited the most pronounced inhibition of cell proliferation in agreement with the high levels of SO_3_^2−^ (Fig. 4H). Thus, the data suggest that bacterial ASR modulates epithelial cell viability through SO_4_^2−^ metabolites.

### The gut microbiota is the primary contributor to serum H2S levels in the DSS-induced mouse colitis model

**Fig. 5:**
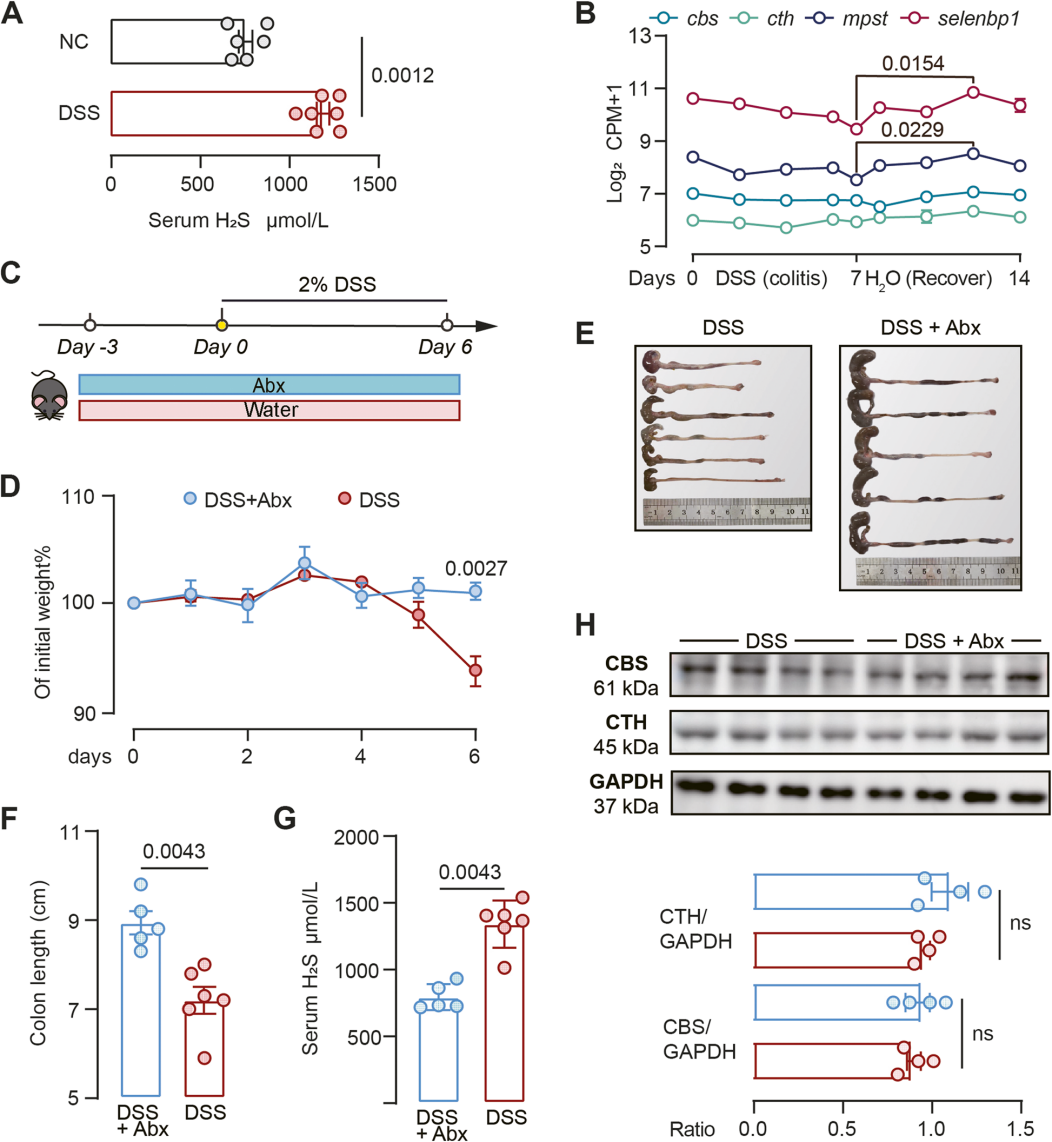
DSS-induced colitis is associated with increased exogenous H_2_S generation. **A** Serum H_2_S level of mice in normal control (NC) and DSS-treated group. Each dot represents an individual mouse. NC, mice were on water (n = 6–7). **B** The expression levels of *cbs*, *cth*, *mpst*, and *selenbp1* in murine colonic tissues were assessed during the administration of DSS and the subsequent recovery period (GSE131032, n = 2 − 3). P-value was determined by nonparametric one-way ANOVA analysis with Dunn’s multiple comparisons. See Fig. 2 for gene full names. **C** Schematic diagram showing the experimental design, timeline of mouse models and sampling strategy. **D** Relative body weight of mice receiving DSS with antibiotics (DSS + Abx) and without (DSS) as shown in Fig B. n = 5–6. Significance was measured with two-way ANOVA with Tukey's multiple comparisons. **E–G** Colonic morphologies (**E**), colon length (**F**) and serum H_2_S level (**G**) of mice under different treatments. **H** Cystathionine beta-synthase (CBS) and cystathionine γ-lyase (CTH) protein levels were analysed by western blotting in mouse colon epithelial tissues. n = 4. Nonparametric Mann–Whitney test was used for non-pairwise comparisons. Each dot represents an individual mouse

Colitis, a key component of IBD, is frequently studied using murine models. One widely employed method to induce colitis in these models is the administration of dextran sodium sulfate (DSS) via drinking water. Our initial objective was to determine if H_2_S production is linked to the DSS-induced colitis model. We found a significant increase in serum H_2_S levels in mice received DSS compared to vehicle controls (Fig. 5A), suggesting that serum H_2_S is associated with DSS-induced colitis.

Although a previous study suggested that germ-free mice exhibit reduced plasma H_2_S levels [42], the specific contribution of the gut microbiota to systemic H_2_S levels in the context of DSS-induced colitis remained unknown. To illuminate the link between elevated serum H_2_S and gut microbes, we performed two studies. First, we utilized a publicly available colonic tissue transcriptomic dataset from mice undergoing DSS-induced colitis, followed by a tissue regeneration phase (GSE131032) [43]. During the colitis and recovery stages, the expression of *cbs* and *cth* genes remained stable, while *mpst* and *selenbp1* expression displayed a decreasing trend during colitis, followed by a slight elevation during the recovery stage (Fig. 5B). This suggested that endogenous H_2_S production remained consistent or even decreased during DSS-induced colitis, hence the rise in serum H_2_S observed is probably from gut microbiota. Second, we administered broad spectrum antibiotics (Abx) to mice in the DSS-induced model (Fig. 5C), and observed a significant reduction in serum H_2_S levels and alleviated DSS-induced colitis, as evidenced by weight and colon length measurements (Fig. 5D-G). Given mRNA levels of *cbs* and *cth* remained stable throughout the DSS-induced colitis and recovery stages (Fig. 5B), we further examined protein levels of CBS and CTH in Abx-challenge mice experiment, and observed no significant difference between the two groups (Fig. 5H). Collectively, these findings provide compelling evidence that the gut microbiota plays a central role in the elevation of systemic H_2_S levels in the DSS-induced colitis model. Therefore, we utilized this model to investigate the causal relationship between microbial ASR pathway and colitis in vivo.

### The gut bacterial ASR pathway contributes to sulfide generation derived from dietary sulfate

Diet plays a pivotal role in shaping the composition and metabolic activity of the gut microbiota. While prior research mainly concentrated on organic sulfur compounds from dietary proteins, the role of inorganic sulfur (SO_4_^2−^) remains understudied [5, 27]. Carrageenan, a common sulfated polysaccharide food additive, is linked to UC relapse risk and can induce intestinal inflammation in animal model [44, 45]. We hypothesized that gut microbiota-mediated carrageenan degradation and subsequent H_2_S production might contribute to its pro-colitis effects.

**Fig. 6:**
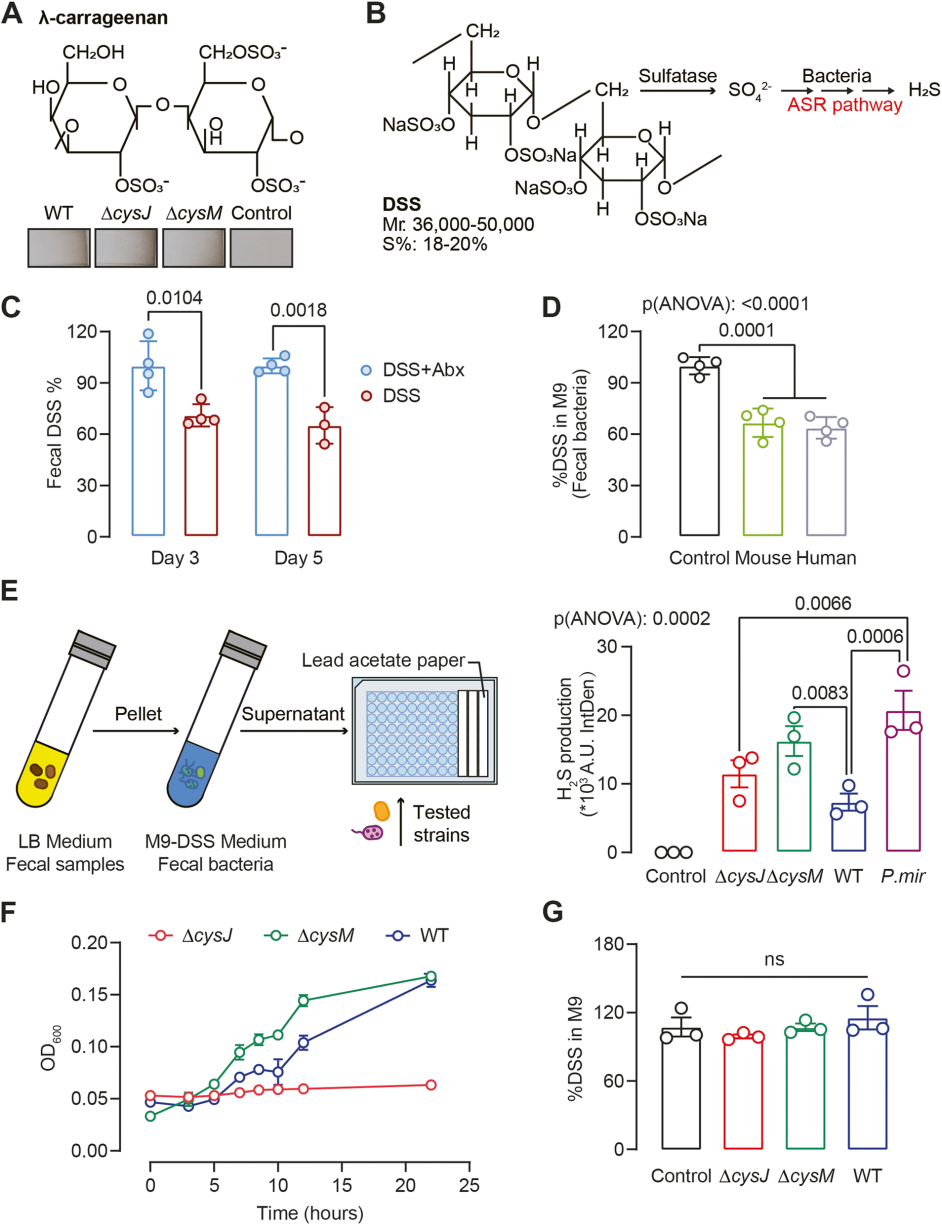
Gut bacterial ASR pathway contributes to H_2_S generation derived from dietary sulfated polysaccharide. **A** *E. coli* WT and mutant strains formed H_2_S from λ-carrageenan. Molecular formula of λ-Carrageenan (above). Representatives of H_2_S detection using lead acetate strip after growing *E. coli* WT and mutant strains in M9 medium supplemented with 1% λ-Carrageenan for 16 h aerobically (below). **B** Schematic outlining H_2_S production from DSS via bacteria. **C** DSS residue rate in mouse feces compared with abx-treated group in day3 and day5. Mice treatment was described in Fig. 5C. Unpaired t-test was used for non-pairwise comparisons. **D** DSS degradation by fecal flora in mice and humans was tested in M9 supplemented with 1% DSS as the sole sulfur source. Medium without bacteria inoculation was used as controls. **E** Flow chart of H_2_S production from DSS test (left). H_2_S production analysis by *E. coli* and *P. mirabilis* strains was examined using lead acetate strip (right). **F** Growth of *E. coli* WT and mutant strains on M9 supplemented with DSS as the sole sulfur source. **G** DSS degradation by *E. coli* WT and mutant strains was tested in M9 supplemented with 1% DSS as the sole sulfur source. Medium without bacteria inoculation was used as controls. Values are Mean ± SEM from at least three independent experiments. P-value was determined by ordinary one-way ANOVA analysis with Tukey's multiple comparisons

To test this hypothesis, we initially cultured *E. coli* WT and mutant strains in M9 medium supplemented with λ-carrageenan as the sole sulfur source, owing to its high sulfur content (32–39%, Fig. 6A). Surprisingly, both WT and mutant strains demonstrated H_2_S production (Fig. 6A), which contrasted with previous findings that *∆cysJ* mutant couldn’t grow on inorganic SO_4_^2−^. Given that carrageenan is a biopolymer derived from red algae, it likely contains trace amounts of organic sulfur compounds that can be utilized by *∆cysJ* mutant. As a result, we transitioned to DSS, a synthetic sulfated polysaccharide with approximately 18–20% sulfur content (Fig. 6B), which as mentioned earlier, is a commonly used as inducer in murine colitis models [46].

We first tested whether the gut microbiota was involved in DSS degradation. Abx-treated mice exhibited significantly higher fecal DSS levels compared to vehicle control mice, suggesting active DSS degradation by gut microbiota in vivo (Fig. 5C, 6C). Ex vivo experiments with mouse and human stool samples showed about 35% of the DSS was consumed after overnight incubation (Fig. 6D), confirmed microbiota-mediated DSS degradation.

We postulated that DSS degradation releases SO_4_ [2−], which are subsequently assimilated by bacteria employing the ASR pathway. To test this hypothesis, we initiated an experiment involving 1% DSS incubation with human/mouse fecal cultures for 16 h, followed by supernatant collection, and subsequent inoculation with *E. coli* WT and mutant strains (Fig. 6E). As anticipated, *E. coli* WT and mutant strains formed H_2_S in the presence of pre-incubated DSS. *E. coli ∆cysM* mutant produced higher levels of H_2_S than WT and the *∆cysJ* mutant, although statistic significance was not attained (Fig. 6E). *P. mirabilis*, which carries the *asr*-gene cluster, generated H_2_S as well (Fig. 6E, Supplementary dataset 4). Direct culture of *E. coli ∆cysJ* mutant in M9 medium with DSS as the sole sulfur source did not yield growth (Fig. 6F). Although *E. coli* WT and *∆cysM* mutant grew on DSS as the sole sulfur source, they showed low utilization and negligible DSS degradation, emphasizing metabolic cross-feeding among bacterial species for efficient sulfated polysaccharide metabolism (Fig. 6F-G).

### The ASR pathway modulates DSS-induced colitis in vivo

**Fig. 7:**
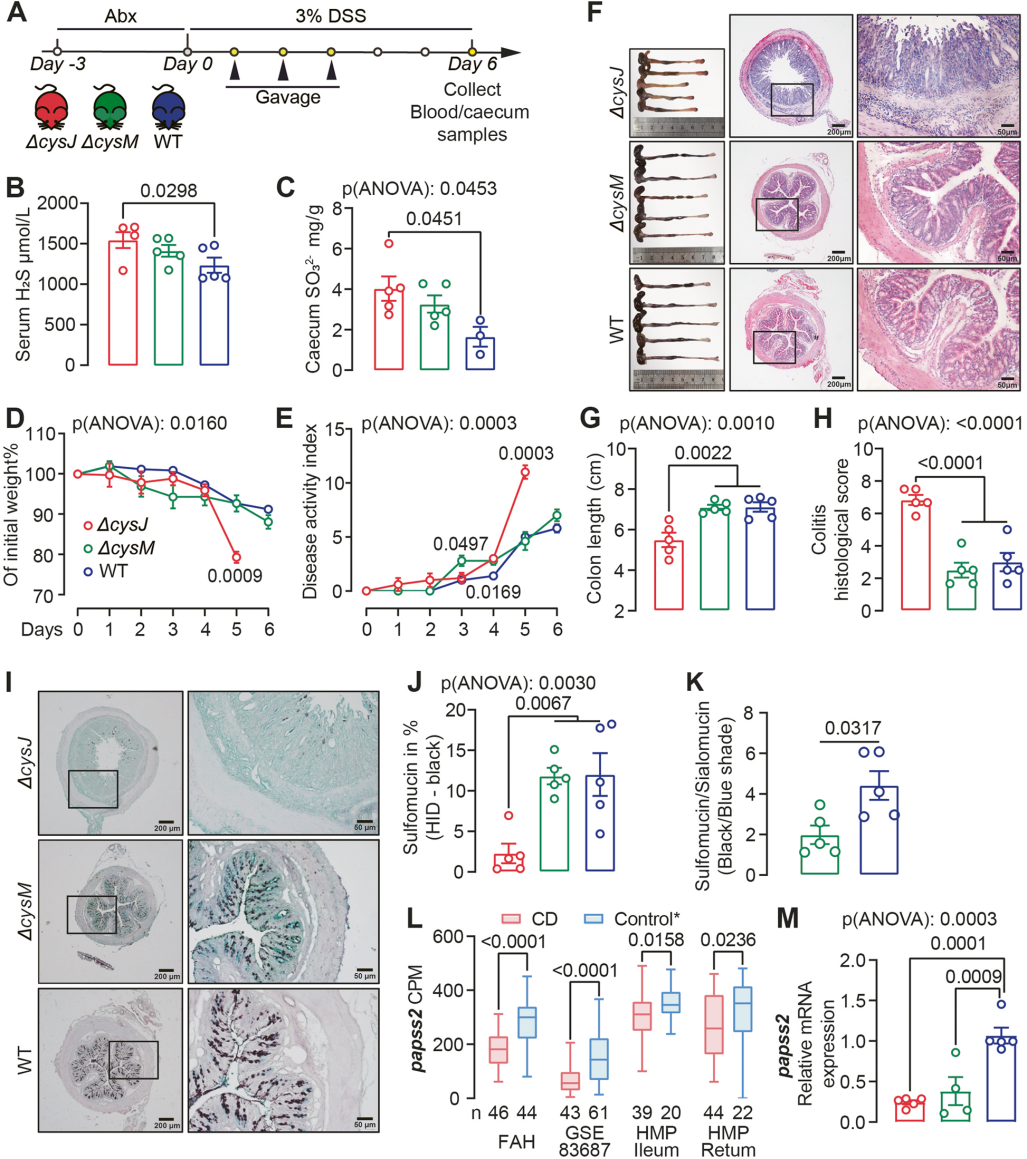
*E. coli* ASR pathway modulates intestinal sulfation and DSS-induced colitis in vivo*.* **A** Schematic diagram showing the experimental design, timeline of mouse models and sampling strategy. **B**,**C** Serum H_2_S and caecum content SO_3_^2−^ levels in mice colonized with *E. coli* WT, *∆cysJ*, and *∆cysM* mutant strains. n = 3–5. Two cecum samples from the WT group were contaminated and excluded from the analysis. **D** Body weight was tracked after DSS administration. n = 5. **E** Disease activity over the duration of the experiment. **F** Colonic morphologies and representative H&E-Stained mouse colon sections at the termination of the experiment on day 6. **G**, **H** Colon length (**G**) and histological assessment of disease severity (**H**). **J** Representative micrographs of High-Iron Diamine-Alcian Blue (HID-AB) staining in mouse colon sections. Sulfomucin is stained black/brown and sialomucin is stained blue. **K-L** Sulfomucin (**K**) and Sulfomucin/Sialomucin (**L**) area ratio among *∆cysJ*, *∆cysM* and WT groups. **M** The box-whiskers plot showed abundance of *papss2* from CD and control subjects from FAH-SYSU, GSE-83687 and HMP cohorts. CPM, copy per million. The highest and lowest values are denoted by the upper and lower extremities of the vertical line, respectively, while the median is represented by the central horizontal line. * Refer to Fig. 2B for details regarding the control groups in each cohort. **N** Relative mRNA levels of *papss2* in the colonic tissue of the mice shown in Fig. 7A. Significance was measured with ordinary one- or two- way ANOVA analysis with Tukey's multiple comparisons. Nonparametric Mann–Whitney test was used for non-pairwise comparisons. Data shown as mean ± SEM. Each point represents an individual mouse

SPF mice received Abx-cocktail were subsequently inoculated with *E. coli* WT, *∆cysJ* and *∆cysM*, then subjected to DSS administration (Fig. 7A). Mice colonized with the *E. coli ∆cysJ* mutant exhibited elevated serum H_2_S and fecal SO_3_^2−^ levels associated with more severe disease phenotype evidenced by a greater body weight loss, a worsening of disease activity, and more severe intestinal inflammation characterized by increased mucosal erosion, crypt destruction and inflammatory cell infiltration in the colon (Fig. 7B-H). The heightened serum H_2_S levels may be ascribed to the degradation of organic sulfur compounds within the gastrointestinal tract, such as L-cysteine, catalyzed by *E. coli ∆cysJ*. We found a significant reduction in plasma levels of ursodeoxycholic acid (UDCA), α- and ω-muricholic acid (MCA), and an increase in cholic acid-7-sulfate (CA-7S) in mice colonized with the *∆cysJ* strain (Fig. S4A), indicating that alteration of *E. coli* ASR pathway strongly influenced the bile acid profile in mice.

While *∆cysM* and WT-colonized mice exhibited similar disease severity, serum H_2_S and fecal SO_3_^2−^ levels, a significant difference in colonic mucin composition was observed. The colonic mucus layer, essential for maintaining homeostasis between resident microbiota and underlying immune cells, is primarily composed of acidomucins, broadly categorized as sialomucins or sulfomucins depending on the presence of sialic acid or sulfate groups [47]. Colonic tissues from *∆cysM*-colonized mice showed a reduced sulfomucin:sialomucin ratio in (Fig. 6I-K), indicating compromised host sulfation than WT mice. Intestinal sulfation, crucial for colitis protection, is dependent on the host PAPS synthase 2 (PAPSS2), which is central in generating PAPS, the universal sulfonate donor for sulfation [41]. Our analysis of transcriptomic data from the FAH-SYSU, HMP and GSE83687 cohorts indicated a significant decrease in colonic *papss2* gene expression in actively inflamed CD patients compared to non-disease and non-IBD controls (Fig. 7L). Mice deficient in *papss2* have been previously demonstrated to manifest reduced intestinal sulfomucin content, rendering them susceptible to DSS-induced colitis [41]. Therefore, we reasoned that reduced host sulfate in *∆cysM*-colonized mice were due to the downregulation of *papss2*. Indeed, we observed decreased colonic mRNA expression of *papss2* in both *∆cysJ* and *∆cysM* groups compared to the WT group, as confirmed by real-time PCR analysis (Fig. 7M).

## Discussion

The burden of IBD, which encompasses conditions like CD and UC, is substantial and often leads to hospitalizations and surgical interventions [1]. Current treatments primarily target host inflammatory pathways using non-specific immunosuppressive agents, which can pose significant risks and may not always be effective, necessitating the exploration of alternative approaches [48]. Mounting evidence suggests that an imbalance in H_2_S production, either insufficient or excessive, can act as an environmental trigger for CD [29, 49]. Studies have shown that the administration of H_2_S donors can suppress the expression of proinflammatory cytokines and ameliorate colitis in murine models [12, 50]. This raises the possibility that modulating H_2_S concentrations in the gut lumen could be an exciting therapeutic strategy for treating CD [51]. However, research into this potential link between H_2_S and CD has been hindered by a limited understanding of sulfur metabolism within the human gut.

To address this knowledge gap, we conducted a comprehensive investigation into the functional capacity of both the gut microbiota and host in H_2_S production. Our findings suggest that microbial sulfur metabolism within the human colon is more complex and widespread than previously recognized. We analyzed metagenomic data from independent IBD cohorts and found that CD is associated with an increase in microbial generation through ASR pathways, as evidenced by the increased abundance and prevalence of *asr*-associated genes. Ex vivo fecal culture confirmed ASR-mediated H_2_S generation is more functionally active in stool samples from CD patients. This aligns with the “oxygen hypothesis”, which suggests that chronic inflammation of the intestinal walls leads to an increased release of hemoglobin carrying oxygen and reactive oxygen species into the intestinal lumen [52]. This process creates a microenvironment that favors facultative anaerobes. Using *E. coli* as the model organism, we generated *∆cysJ* and *∆cysM* mutants deficient in the ASR pathway. We conducted in vitro and in vivo studies to validate that the bacterial ASR pathway modulates cell viability, host sulfate homeostasis, and colitis pathogenesis. Our investigation has brought into focus the pivotal role played by ASR pathway in reshaping the utilization of L-cysteine and generation of H_2_S. The deletion of *cysJ* gene in *E. coli* amplifies H_2_S production from L-cysteine. The heightened metabolism of L-cysteine by gut microbes and increased abundance of *cysM* has recently been associated with CRC [34]. We noticed that *asrABC* is enriched in CD subjects, warranting further investigation into its potential association with CD.

In contrast to previous research, our findings suggest that the DSR pathway is unlikely to be the primary contributor to the elevated fecal microbial sulfidogenic capacity in CD. Prior research on exogenous H_2_S generation primarily centered on DSR, based on the culturing and sequencing of *Desulfovibrio* genus, sulfate-reducing bacteria frequently found in the human and animal gut [28, 53]. However, Anantharaman et al. [54] revealed that *dsrAB*-mediated dissimilatory sulfur metabolism is predicted in a much broader diversity of bacterial and archaeal groups than previously recognized, primarily due to horizontal gene transfer, such as *Bilophila wadsworthia*, an opportunistic pathogen inhabiting the gut. Consequently, it is more reasonable to predict DSR-mediated H_2_S generation based on *dsr*-gene cluster quantification, rather than relying solely on *Desulfovibrio* quantification. The gut microbiota not only generates H_2_S but also has the capability to oxidize it using sulfide:quinone oxidoreductase and persulfide dioxygenase [55, 56]. Further studies are warranted to gain a comprehensive understanding of gut microbiota sulfur metabolism.

Analysis of intestinal biopsy transcriptomic data from multiple IBD cohorts has unveiled a compromised endogenous sulfidogenic capacity in CD patients. This is evident from the downregulation of key genes, specifically *cbs*, *cth*, *mpst* and *selenbp1*. Severe CD manifestations in a child with *cbs* deficiency has been reported [57]. Reduced expression of *cbs mpst* and *selenbp1* has been linked to the exacerbation of inflammation-induced intestinal barrier injury in UC and CD [58–60],. Animal studies have provided additional evidence highlighting the critical role of endogenous H_2_S generation in colitis. MPST^−/−^ and MPST^±^ mice exhibit exacerbated DSS-induced colitis [59]. Inhibition of endogenous H_2_S synthesis through the use of CBS and CTH inhibitors, such as β-cyanoalanine, propargylglycine, and O-carboxymethyl-hydroxylamine hemihydrochloride, has been demonstrated to worsen colitis in mouse model [12]. A correlation has been identified in immune deficiency and impaired H_2_S synthesis. Numerous studies have demonstrated that IL-10 plays an essential role in maintaining mucosal immunological tolerance in patients with IBD [61]. Flannigan et al.'s study with IL-10-/- mice, which spontaneously develop colitis, revealed a significant impairment in colonic H_2_S synthesis. This impairment was reversed by the administration of recombinant IL-10, confirmed an interplay between IL-10 and H_2_S synthesis [62].

Considerable efforts are underway to investigate the potential contributions of dietary factors to the pathophysiology of IBD [63, 64]. The interplay of genetic, environmental, microbial, and immunological factors makes diet a crucial aspect of IBD etiology [65]. Dietary sulfur intake, primarily from inorganic sulfate and sulfur-containing amino acids (SAAs) such as methionine, cysteine, and taurine, plays a significant role [5, 27]. However, estimations of dietary sulfur content often fail to account for sulfur-containing food modifiers or additives, such as carrageenan and sulfiting agents (*e.g.*, potassium bisulfate, sodium bisulfate) [66]. Daily intake of inorganic sulfate is estimated to range from 1.5 to 16.0 mmol [66]. Interestingly, fecal sulfate (and sulfide) excretion is minimal compared to dietary intake, suggesting that sulfate is actively removed from the fecal stream during passage through the gut by both host and gut microbiota [67]. Carrageenan, a sulfated polysaccharide, contains approximately 15% to 40% sulfur, depending on the specific type of carrageenan (*e.g.,* kappa, iota, lambda) and the seaweed species used for extraction [68]. Carrageenan is widely used as a food additive in the Western diet, and its consumption has substantially increased over the past 50 years, paralleling the rising prevalence of IBD [69]. Successful dietary interventions that induced CD remission have excluded processed foods containing carrageenan, further supporting the notion that carrageenan may trigger or exacerbate inflammation in IBD [70]. In animal models, carrageenan administration consistently induces intestinal ulcerations resembling human IBD histopathologically [45]. We reasoned that SO_4_^2−^ released during carrageenan degradation are utilized by gut microbes, contributing to IBD pathogenesis. To investigate, we used synthetic sulfated polysaccharide DSS due to carrageenan's organic sulfur contaminants. The DSS-induced colitis model is known for consistently mimicking epithelial damage seen in IBD, underscoring the complex interplay between dietary elements, gut microbiota, and disease pathogenesis [46]. DSS mouse colitis model is known for its variability even among genetically identical mice and across different mouse facilities. A recent study found that gut microbiota plays a significant role in driving this variability within the model [71].

Through in vivo and ex vivo studies, we've demonstrated that the gut microbiota can degrade DSS, releasing SO_4_^2−^ that fuel bacterial ASR pathways. This alteration in microbial sulfur metabolism ultimately modulates disease severity. Our study highlights the crucial role of gut microbial ASR metabolism in dietary sulfate metabolism and susceptibility to colitis. It's important to acknowledge the significant daily intake of inorganic sulfate and the potential exacerbation of microbial H_2_S production by carrageenan used as food additives in processed foods, which can lead to mucosal damage. Furthermore, it is worth noting that CD pathogenesis is unlikely to be solely attributed to the presence and activities of single species. Genes associated with the ASR pathway exhibited varying distribution patterns among human bacteria. Additionally, in DSS degradation, mouse and human fecal microbial communities are more efficient than *E. coli* monocultures, suggesting cross-feeding among different bacterial species for the efficient metabolism of sulfated polysaccharides.

In summary, our study reveals the extensive diversity of microbial sulfur metabolism pathways. These findings highlight the association between CD and reduced endogenous H_2_S production alongside increased gut microbial H_2_S generation, primarily via the ASR pathway. Microbial ASR-mediated dietary sulfate metabolism emerges as a crucial factor in colitis. Thus, it is essential to maintain the homeostasis of microbial assimilatory sulfate reduction. Further research is needed to elucidate the regulation of the cysteine regulon and its impact on CD. Our research sheds light on the complex interaction between diet, the gut microbiota, and inorganic sulfate metabolism, highlighting their potential as promising therapeutic targets for managing CD.

## Materials and Methods

### Human subjects

All study protocols abided by the Declaration of Helsinki principles and were approved by Ethical Committees of the First Affiliated Hospital of Sun Yat-sen University. Intestinal biopsies and stool specimens were collected as part of the FAH-SYSU cohort study (2016[113]). Subject stool samples were collected at the FAH, SYSU gastroenterology clinic and stored at -80 °C immediately. For culturing assays, fecal samples were collected and diluted to make a 10% (w/v) fecal slurry by resuspension of the feces in 10% (w/v) glycerol solution, and aliquots were stored in cryogenic vials at -80 °C until use. The exclusion criteria applied to all groups were as follows: recent (< 3 months prior) use of any antibiotic therapy, current extreme diet (*e.g.*, parenteral nutrition or macrobiotic diet), known history of malignancy, current consumption of probiotics, any gastrointestinal tract surgery leaving permanent residua (*e.g.*, gastrectomy, bariatric surgery, colectomy), or significant liver, renal, or peptic ulcer disease.

### Analyses of *asr*- and *dsr*-associated genes in Human Microbiome project (HMP) references genomes

HMP references genomes (1635 genomes as of June 30, 2023) were selected and analyzed through the IMG program on the Joint Genome Institute website (https://img.jgi.doe.gov/) [72]. The functions (Supplementary dataset 1) were used to carry out a “Function Profile” against all selected reference genomes to identify those carrying *asr*- and *dsr*-associated genes. Hits were manually inspected. Genomes carrying sulfidogenic gene(s) were selected to generate a phylogenetic tree using phyloT (https://phylot.biobyte.de/) based on NCBI taxonomy and visualized using iTOL [73]. Genome and gene IMG ID are available in Supplementary dataset 4.

### Metagenomic data analysis

We used ShortBRED [31] to accurately profile the abundance of genes involved in the H_2_S generation in metagenomes sourced from the FAH-SYSU (BioProject: PRJNA793776) [74] and PRISM (BioProject: PRJNA400072) [3] datasets. We initially compiled a set of identified bacterial sulfidogenic genes as our query sequences (Supplementary dataset 6). Subsequently, ShortBRED-Identify was employed to generate markers for these key bacterial sulfidogenic gene sequences using UniRef90 (May, 2023) as a reference list with an 85% cluster ID threshold. These markers were applied in ShortBRED-Quantify to assess gene abundance in paired metagenomes, which had previously undergone quality control via the KneadData workflow (http://huttenhower.sph.harvard.edu/kneaddata). The output from ShortBRED-Quantify was expressed as reads per million reads per kilobase million (RPKM).

### Cultivation of wild type bacteria and mutants

*Escherichia coli* MG1655 wild type, mutants (Δ*cysJ and* Δ*cysM*) and *Proteus mirabilis* ATCC 29906 were generally cultivated in Luria broth (LB) containing tryptone (10 g· L ^−1^), yeast extract (5 g· L ^−1^) and NaCl (10 g· L^−1^). To characterize the growth of *E. coli* wild type and mutant strains, they were cultivated in 5 mL LB overnight at 37 °C in a shaking incubator (250 rpm) and the pellet was collected by centrifugation at 3,000 × g for 10 min. Cell pellets were then washed and re-suspended in fresh M9 media (inoculum size 1:20, *v/v*). The defined M9 medium contained NaCl (0.5 g·L^−1^), KH_2_PO_4_ (3 g·L^−1^), Na_2_HPO_4_·12H_2_O (6 g·L^−1^), NH_4_Cl (1 g·L^−1^), MgCl_2_ (95 mg·L^−1^), CaCl_2_ (11.1 mg·L^−1^) and glucose (0.1%, w/v). 1 mM Na_2_SO_4_, Na_2_S_2_O_3_, L-cysteine or DSS was used as sole sulfur source. 200 µL samples were collected from each tube at the indicated time points, and their optical density at 600 nm was measured in flat-bottom 96-well plates (200 µL per well). Sulfite in the supernatant was quantified as described in Sulfite Quantification section.

### Allele-exchange mutagenesis of *ΔcysJ* and *ΔcysM* in *E. coli* MG1655

DNA fragments (~ 1 kb) corresponding to the upstream and downstream regions of the target gene were amplified and a subsequent overlap PCR was used to fuse the two fragments which were then ligated into suicide plasmid harboring kanamycin resistance cassette, *oriT* (mob), *sacB* counter selection marker and R6K origin of replication using the In-Fusion HD Cloning kit (Clontech). The ligated suicidal plasmid (pKmobSac) was transformed into the donor strain, *E. coli* S17 λpir. In parallel, *E. coli* MG1655 was transformed with a temperature-sensitive ampicillin-resistance plasmid carrying oriR101 origin of replication (p101-Amp). The suicidal plasmid (pKmobSac) was then transformed into *E. coli* MG1655 through conjugation and the resulted conjugants were screened at room temperature on LB agar plates containing ampicillin at 100 ng/µL (to select against *E. coli* S17 donor cells) and kanamycin at 50 ng/µL. One single-crossover integrant was then selected and re-streaked on LSW-Sucrose agar plate (tryptone 10 g/L, yeast extract 5 g/L, glycerol 5 mL/L, NaCl 0.4 g/L, sucrose 100 g/L and agar 20 g/L) [75] supplemented with ampicillin at 100 ng/µL to select for the correct double cross-over mutants. One mutant was then selected, re-streaked, and confirmed for the loss of the conjugated plasmid through Sanger sequencing and its ability to grow in presence of ampicillin but not kanamycin. The knockout *E. coli* MG1655 mutants were then cured from p101-amp plasmid through growing at 37 °C.

### H_2_S quantification

Plasma H_2_S levels were quantified using a modified methylene blue method. In brief, ZnAC was added to 100 μL of plasma samples to precipitate H_2_S, HS^−^, S^2−^, and plasma proteins. Subsequently, the ZnS pellet was re-dissolved by adding 130 μL of 2% N, N-dimethyl-p-phenylenediamine and 130 μL of 20% trichloroacetic acid. Methylene blue formation was initiated by addition of FeCl_3_·6H_2_O and quantified at 665 nm using a spectrophotometer. Microbial culture H_2_S levels were quantified by Modified Sulfur, Indole, Motility (SIM)-medium and Lead Acetate Test Strip. See online supplemental material for further details.

### Dextran sulfate quantification

To measure DSS concentration in mouse fecal pellets, Sample aliquots (50 µL) were injected onto a size exclusion column (SEC-150, 3 μM, 7.8 × 300 mm, Welch, Cat # 00237–21052) and eluted at a flow rate of 1.5 mL/min. The mobile phase consisted of 25 mM KH_2_PO_4_, 25 mM K_2_HPO_4_·3H_2_O, 50 mM KCl, and 10% ethanol. The eluent passed through a post-column derivatization instrument (LABRAT, LYM-1060), where it mixed with a 10 μg/mL dimethylene blue zinc chloride double salt (DMB, Sigma, 34108) delivered directly by pump A connected to the online mixer. Detection was performed using a VWD detector at 530 nm wavelength, with data collected via OpenLAB CDS chromatography data software (1260 Infinity II, Agilent, Hong Kong, China).

### Sulfite quantification

150 μL of culture supernatant or caecum slurry extract was mixed with 350 μL distilled water and 10 μL 10 M NaOH. Sulfite was quantified using the Total Sulfite Assay Kit (JC-HX-04, HK, China) based on the Pararosaniline Method, following the manufacturer's instructions. The reaction formed a purple-red complex, pararosaniline methylsulfonic acid, which exhibited maximal absorption at 550 nm, and absorbance was measured after a 10-min incubation using a plate reader (UV-2450, SHIMADZU, Japan).

### Cell culture and viability assay

The NCM460 human colon epithelial cell line (RRID: CVCL_0460) was maintained in RPMI 1640 basic medium (Gibco, Thermo Fisher Scientific, MA, USA) supplemented with 10% fetal bovine serum (FBS; Thermo Fisher Scientific, Waltham, MA, USA), 100 µg/mL penicillin G, and 100 µg/mL streptomycin sulfate (Invitrogen, Carlsbad, CA, USA). Cells were cultured at 37 °C in a 5% CO_2_ humidified incubator.

Cell viability was analyzed by live/dead staining or Cell Counting Kit-8 (CCK8, GLPBIO, GK10001) according to the manufacturer's instructions. For live/dead staining, NCM460 cells (4 × 10^3^/well) in 96-well microplates were cultured for 48 h in 100 μL of 1640 medium. After washing with PBS, cells were prepared for bacterial co-culture. *E. coli* strains were cultivated overnight at 37 °C in LB, washed in sulfur-free M9 media, and resuspended in M9 medium with L-cysteine or Na_2_S_2_O_3_ as the sole sulfur source. Cells were co-cultured with these bacteria for 2 h (150 μL bacterial culture per well). Afterward, cells were stained with 30 μL calcein-AM/PI working solution (2 μM calcein-AM and 4.5 μM propidium iodide) at 37 °C for 20 min, followed by fixation with 0.4% polyformaldehyde. Quadruplicate experiments were conducted, and images of cells were acquired and analyzed using an Olympus IX83 fluorescence microscope (Olympus, Tokyo, Japan). All experiments were performed in quadruplicate. Positive cell percentages and average fluorescence intensity were determined using Image-Pro Plus 6.0. For CCK8 assay, NCM460 cells (5 × 10^3^/well) were cultured overnight in 96-well microplates and treated with 50 μL of bacterial supernatants from M9 medium cultures with 2 mM Na_2_SO_4_ as the sole sulfur source. After a 6-h incubation, cells were washed, incubated with 100 μL of RPMI 1640 Medium plus 10 μL CCK-8 reagent, and absorbance at 450 nm was monitored. All experiments were performed in six replicates and blank wells without cells served as controls.

### Animal studies

Male SPF C57BL/6 mice (6–8 weeks) were maintained on a standard normal rodent diet (Synergy Bio, AIN-93 M, Jiangsu, China). All the mice used in this study were bred and raised in the animal facility of the First Affiliated Hospital of Sun Yat-sen University. Mice (n = 6) received antibiotic cocktail (Abx) [76] for 5 days prior to 2% DSS ad libitum in drinking water for 6 days. Mice (n = 6) without Abx pre-treatment was used as vehicle control. Fecal samples from days 3 and 5 were processed by mixing 0.05 g of fresh feces with 500 μL PBS, vortexed for 3 min, and centrifuged at 14,000 × g for 10 min. The supernatant was used for DSS quantification as described in DSS quantification section. For *E. coli* gavage experiment, mice received Abx-water were subsequently administered *E. coli* wild type and mutant strains via oral gavage at a dose of 1.0 × 10^9^ cfu/200 µL each. DSS was supplied in drinking water at 3% (w/v) for 6 days. Caecum was harvested for sulfite quantification as described in Sulfite Quantification section.

Mice were monitored daily for body weight, stool consistency and stool bleeding. Mice were euthanized by cervical dislocation after 5–6 days of DSS treatment. Colon samples were collected for histological, western blot and qRT-PCR analysis. See online supplemental material for further details.

### Bacterial H_2_S Production from λ-Carrageenan and DSS

Bacteria from the LB start culture were harvested, washed, and inoculated in 5 mL sulfur-free M9 medium with 1% λ-carrageenan (inoculum size 1:200, *v/v*), incubated overnight at 37 °C while a suspended lead acetate strip monitored H_2_S generation. Medium without inoculum was set up as a negative control.

To assess DSS degradation, mouse and human fecal samples (2 CD subjects and 2 healthy individuals) were cultured in 10 mL LB medium (OD_600_ = 1.0). Bacterial pellets were resuspended in 5 mL sulfur-free M9 medium with 1% DSS. DSS levels in supernatants were measured via SEC-HPLC, and H_2_S production was monitored with suspended lead acetate strips.

To investigate H_2_S production from DSS by *E. coli* WT, mutants, and *P. mirabilis*, cultures were grown overnight in 10 mL LB medium at 37 °C, 250 rpm. After harvesting, cells were resuspended in 1 mL sulfur-free M9 medium. Pre-incubated DSS medium was prepared using supernatant from M9 medium with 1% DSS and cultures from two CD subjects, as described earlier. Subsequently, 200 µL of this pre-incubated DSS medium and sulfur-free M9 medium with 1% DSS were inoculated with 50 µL of bacterial cultures. Incubation was conducted at 37 °C in a 96-well plate with lead acetate strips for sulfide quantification (see Sulfide Quantification, Lead Acetate Strip section).

### Statistical analysis

Statistical analyses were performed with Prism v.8.0 (GraphPad). For two-group comparisons, the statistical significance was determined by unpaired t test or nonparametric Mann–Whitney test as indicated. Multiple group comparisons were made by ANOVA for most of the studies as indicated. Each data point denotes an individual human subject, animal, or biological replicate.

### Supplementary Information

Supplementary Material 1

Supplementary Material 2

## Data Availability

All study data are included in the article and/or SI Appendix. Data are available in a public, open access repository. Gene expression profiling by high-throughput sequencing data have been deposited in Gene Expression Omnibus accession no. (GSE83687 and GSE131032) and Biostudies accession no. (E-MTAB-54674). Metagenomic sequences for the PRISM is available via SRA with BioProject number PRJNA400072. Raw metagenomic data of the FAH-SYSU cohort were deposited in the NCBI public repository (Bioproject #PRJNA793776). HMP IBD metagenomics and transcriptomic data can be accessed at https://ibdmdb.org/tunnel/public/summary.html. The raw RNA-seq data of FAH-SYSU cohort have been deposited in the Genome Sequence Archive [77] in National Genomics Data Center [78], China National Center for Bioinformation/Beijing Institute of Genomics, Chinese Academy of Sciences (GSA-Human: HRA007763) that are publicly accessible at https://ngdc.cncb.ac.cn/gsa-human. All plasmids, bacterial mutant strains, and reagents generated in this study are available from the lead contact upon completing Material Transfer Agreement. Any additional information required to reanalyze the data reported in this paper is available from the lead contact upon request.
