# Supplementary material for: Microbial assimilatory sulfate reduction-mediated H2S: an overlooked role in Crohn’s disease development
Source: Microbiome. 2024 Aug 16;12:152. doi: 10.1186/s40168-024-01873-2 (PMC11328384; doi:10.1186/s40168-024-01873-2)
Supplement: Supplementary file 2 — Supplementary Material 1 [file 40168_2024_1873_MOESM1_ESM.docx]

**Supporting Information for**

Microbial Assimilatory Sulfate Reduction-Mediated H_2_S: An Overlooked Role in Crohn's Disease Development

Wanrong Luo^a,b,1^, Min Zhao^c,1,^ Mohammed Dwidar^d,e^, Liyuan Xiang^a^, Yang Gao^b^, Xueting Wu^a^, Marnix H. Medema^f^, Shu Xu^a^, Xiaozhi Li^a^, Hendrik Schaefer^g^, Minhu Chen^a,2^, Rui Feng^a,2^, Yijun Zhu^b,2^

a Department of Gastroenterology, the First Affiliated Hospital, Sun Yat-sen University, Guangzhou, China

b Institute of Precision Medicine, the First Affiliated Hospital, Sun Yat-sen University, Guangzhou, Guangdong, China

c Department of Gastroenterology, Shenzhen No.3 People's Hospital, Shenzhen, Guangdong, China

d Department of Cardiovascular & Metabolic Sciences, Lerner Research Institute, Cleveland Clinic, Cleveland, OH, USA

e Center for Microbiome and Human Health, Cleveland Clinic, Cleveland, OH, USA

f Bioinformatics Group, Wageningen University, Wageningen, The Netherlands

g School of Life Sciences, University of Warwick, Coventry, UK

1 These authors contributed equally

2 These authors contributed equally

Corresponding author:

Minhu Chen, M.D, PhD

Department of Gastroenterology, the First Affiliated Hospital, Sun Yat-sen University

No.58 Zhongshan Er Road, Room 1209; Guangzhou, China 510080

Tel: +86 (20)-87755766

**Email:** [chenminhu@mail.sysu.edu.cn](mailto:chenminhu@mail.sysu.edu.cn)

Rui Feng, M.D, PhD

Department of Gastroenterology, the First Affiliated Hospital, Sun Yat-sen University

No.58 Zhongshan Er Road, Room 1209; Guangzhou, China 510080

Tel: +86 (20)-87755766

**Email:** [fengr7@mail.sysu.edu.cn](mailto:fengr7@mail.sysu.edu.cn)

Yijun Zhu, PhD

Institute of Precision Medicine, the First Affiliated Hospital, Sun Yat-sen University.

No.1 Zhongshan Er Road, Room 1209; Guangzhou, China 510080

Tel: +86 8760 6870 (ext) 1209

020-87755766

**Email:** [zhuyj67@mail.sysu.edu.cn](mailto:zhuyj67@mail.sysu.edu.cn)

**Leading author:** zhuyj67@mail.sysu.edu.cn (Y.Z.)

**This PDF file includes:**

Supporting text

Figures S1 to S4

Legends for Datasets S1 to S7

SI References

**Other supporting materials for this manuscript include the following:**

Datasets S1 to S7

Supporting Information Text

Materials and Methods

**Subhead.** Type or paste text here. You may break this section up into subheads as needed (e.g., one section on “Materials” and one on “Methods”).

**A modified methylene blue assay**

Plasma H_2_S levels were quantified using a modified methylene blue method. 10 μL of 20% ZnAC was added to 100 μL of plasma samples and incubated at room temperature for 30 minutes to precipitate H_2_S, HS^-^, S²-, and plasma proteins. After centrifugation (12,000×g for 10 min), the pellet was re-dissolved in 100 μL of 10M NaOH to dissolve plasma proteins, followed by a second centrifugation and two washes with ddH_2_O. Subsequently, the ZnS pellet was re-dissolved by adding 130 μL of 2% N, N-dimethyl-p-phenylenediamine and 130 μL of 20% trichloroacetic acid. After centrifugation, 250 μL of the supernatant was transferred to a microplate, and 13 μL of 2% FeCl_3_·6H_2_O was added to initiate methylene blue formation, which was quantified at 665 nm using a spectrophotometer, with a standard curve prepared using the NaS standard in a similar manner.

**Modified Sulfur, Indole, Motility (SIM)-medium** The modified SIM medium was composed of Pancreatic digest of casein (20 g·L^-1^), Peptic digest of animal tissue (6.1 g·L^-1^), agar (3.5 g·L^-1^), Fe(NH_4_)_2_(SO_4_)_2_·6H_2_O (0.2 g·L^-1^), with pH adjusted to 7.3±0.2. To assess H_2_S generation, various sulfur sources (1 mM Na_2_SO_4_, Na_2_S_2_O_3_, L-cysteine, or L-Methionine) were added. Cultures in 24-well plates combined 3 mL of SIM medium with 10 μL of bacterial cultures (OD_600_ = 1.0) from LB medium, and were aerobically incubated overnight at 37°C.

**Lead Acetate Test Strip** Overnight bacterial cultures (initially OD_600_ = 1.0 in LB medium) were washed twice with M9 salt solution, diluted 1:200 in 200 μL sulfur-free M9 medium with varied concentrations of L-cysteine, and incubated at 37°C aerobically and anaerobically in 96-well plates. For H_2_S detection in fecal samples, 2 mg of fecal slurry from healthy individuals and CD patients was inoculated into 200 μL Fe^2+^-free SIM medium with 1 mM Na_2_SO_3_, in 96-well plates, and incubated aerobically at 37°C overnight. Lead acetate strips (Macklin, L903523-1EA) were attached to the interior surface of a 96-well plate lid. Each sample was analyzed in triplicate. H_2_S production, indicated by brown spots, was quantified using ImageJ's IntDen function and normalized by subtracting the background.

**Bacterial morphology**

Bacteria pellet was fixed in 2.5% glutaraldehyde in 0.1M phosphate buffer (pH = 7.3) for 30 min at room temperature, then centrifuge and discard supernatants. Pellet was re-suspended in 0.1 M phosphate buffer (pH 7.4), then prefixed with 1% agarose solution. Agarose blocks with samples were post-fixed with 1% OsO_4_ at 0.1 M phosphate buffer at room temperature for 2 h. Prior to analysis, the sample was dehydrated using a rising series of ethanol and acetone. Subsequently, the samples were embedded in acetone/EMBed 812 (SPI, 90529-77-4) for 2 to 4 h at 37°C, and the resin embedding models were polymerized in a 65°C oven for more than 48h. The ultrathin sections (60-90 nm) were affixed to cuprum grids with formvar film on 150-mesh grids and then subjected to staining and examination using transmission electron microscopy (HITACHI, HT7800).

**Proteomics analysis**

Bacterial cultures grown in 40 mL of M9 medium containing 1mM Na_2_S_2_O_3_ were harvested and resuspended in 2 mL Tris-HCl buffer (pH 8.0) for sonication (100 W) on ice. After centrifugation (3000×g, 10 min), supernatants were used for comparative untargeted proteomics analysis. Bacterial cell lysates underwent in solution reduction, alkylation, and tryptic digestion using 0.5 μg trypsin per sample. After overnight incubation, digested samples were centrifuged at 21,000xg for 15 min. Five µg of each digest was dried down, reconstituted in 25 µL of 0.1% formic acid in water, and analyzed by LC-MS/MS using an Orbitrap Exploris 480 with an Easy-nLC1200 HPLC system (ThermoFisher Scientific). Peptide digests were introduced onto a trap column (PepMap C18 2 cm × 75 µm, 100 Å) and subsequently resolved on a Nanoviper reverse phase C18 column (75 µm × 250 mm, 2 µm, 100 Å) and eluted at a flow rate of 0.3 µL/min using mobile phase A (0.1% formic acid in water) and B (0.1% formic acid in 80% acetonitrile). The gradient was increased linearly from 6% to 95%. A data-dependent acquisition method was used that involved full scan MS1 (350-1,500 Da) acquisition in the Orbitrap mass spectrometer at a resolution of 60,000. Protein identification and quantification were achieved by database searching with Proteome Discoverer v2.5.0.400 against *Escherichia coli* K12 complete proteome sequence (http://www.uniprot.org/, December 2022).

**Mouse colitis model**

Male SPF C57BL/6 mice (6-8 weeks) were maintained on a standard normal rodent diet (Synergy Bio, AIN-93M). All the mice used in this study were bred and raised in the animal facility of the First Affiliated Hospital of Sun Yat-sen University.

**Murine antibiotics challenge** Mice were randomly divided into two groups. Mice (n=6) received antibiotic cocktail ^1^ for 5 days prior to 2% DSS *ad libitum* in drinking water for 6 days. Mice (n=6) without antibiotic pre-treatment was used as vehicle control. Fecal samples from days 3 and 5 were processed by mixing 0.05 g of fresh feces with 500 μL PBS, vortexed for 3 min, and centrifuged at 14,000×g for 10 min. The supernatant was used for DSS quantification as described in DSS quantification section.

***E. coli* WT and mutant strains colonization** Mice were randomly allocated into three groups (5 per group) and received oral poorly absorbed broad spectrum antibiotics cocktail (Abx) previously shown for 3 days to suppress gut microbiota^1^. Mice were subsequently administered *E. coli* wild type and mutant strains *via* oral gavage at a dose of 1.0 × 10^9^ cfu/200 µL each at day 0,1,2,4. DSS was supplied in drinking water at 3% (w/v) for 6 days. Fecal samples were collected and stored at −80°C until use. For caecum slurry sulfite quantification, 1 g of caecum contents mixed with 1 mL PBS, vortexed, centrifuged (14,000×g, 10 min), and sulfite was quantified as described in Sulfite Quantification section.

Mice were monitored daily for body weight, stool consistency and stool bleeding. Colitis severity was scored by evaluating these clinical disease activities. The Disease Activity Index (DAI) was determined as previously described^2^. Mice were euthanized by cervical dislocation after 5-6 days of DSS treatment. A 7-mm section of the distal colon, located approximately 1 cm from the rectum, was extracted, fixed in Carnoy's solution (Solarbio, G2310), and prepared for histological analysis.

**Histopathological analysis**

Hematoxylin-Eosin staining utilized the HE Stain Kit (Solarbio, G1120) and was scored according to a previously established system^2^. High Iron Diamine-Alcian Blue staining involved incubation in high-iron diamine solutions for 18 hours and subsequent exposure to alcian blue (pH 2.5) solution for 30 minutes, following established procedures^3^. The quantifications of sulfomucin and sialomucin area per mouse were determine using Image-Pro Plus 6.0 software.

**Western Blot Analysis**

Colon protein samples were prepared by RIPA lysis (EpiZyme, PC101) with a protease inhibitor cocktail (EpiZyme, GRF101) and quantified using a BCA protein assay kit (EpiZyme, ZJ101). Equal protein amounts (25 μg) were separated on a 10% SDS-PAGE gel (EpiZyme, PG212), transferred to a PVDF membrane (Millipore, IPVH00010), and blocked with 5% skim milk for 1 hour at room temperature. The membranes were incubated overnight at 4°C with CBS antibody (1:2000 dilution; GeneTex, GTX113400), CTH antibody (1:3000 dilution; GeneTex, GTX113409-S), and GAPDH (1:3000 dilution; Affinity, AF7021), followed by incubation at room temperature for 1 hour with the HRP-conjugated secondary antibody (1:5000 dilution; Proteintech, PR30011), and blots were developed with ECL detection reagents (EpiZyme, SQ101). Images were captured using a gel imaging system (AmershamTM ImageQuantTM 800), and protein quantification was performed using Image J software to analyze image gray areas.

**Quantitative Real-Time Polymerase Chain Reaction**

Total RNA was isolated employing the Eastep® Super Total RNA Extraction Kit (Promega, LS1040), followed by reverse transcription of the isolated mRNA into cDNA using the Evo M-MLV RT Kit (Accurate Biology, AG11706) following the manufacturer's instructions. The qRT-PCR mixture consisted of 50 ng cDNA, 300 nM forward and reverse primers, and SYBR Green Pro Taq HS Mix (Accurate Biology, AG11719). Amplification was conducted in a QuantStudio Real-Time PCR system (Applied Biosystems). Each sample was done in triplicate. Data were normalized to the expression of the housekeeping gene *gapdh*. The primers are listed 5′–3′ as follows: *gapdh*: F, GTCGTGGATCTGACGTGCC; R, TGCCTGCTTCACCACCTTCT; *papss2*: F, TGGTGCTGGGAAAACAACCA; R, TCCCCGCAGAGAATCCCAG^4^.

**Bile Acids Measurement**

Mice serum samples collected when executed, were then stored at −80 °C. Before processing, serum samples were thawed on ice-bath to diminish sample degradation. An aliquot (20 μL) was mixed with 80 μL LC/MS grade ice-cold methanol containing internal standards (Supplementary dataset 7). Samples were vortexed for 10 min and centrifuged at (18,000×*g*, 20 min, 4 °C). The supernatant was transferred to HPLC vials with glass inserts and kept at −20 °C until analyzed. A serial concentration of standard mixtures of 25 bile acids (BAs) were prepared in methanol and were used to establish calibration curves for quantification. The analysis of all calibrators and samples were performed on ultra-performance liquid chromatography coupled to electrospray ionization tandem mass spectrometry platform (SCIEX Triple Quad™ 7500 LC-MS/MS System). Mobile phase A was water with 0.01% acetic acid, mobile phase B was acetonitrile. Samples were injected (10 μL) and chromatographically separated on a reverse phase column (Phenomenex Kinetex C18, 2.6 μM, 150 mm x 4.6 mm ID), and the target BAs were eluted with a 23–77% gradient of mobile phase B. Analytes were monitored using MRM transition listed in Supplementary dataset 7.


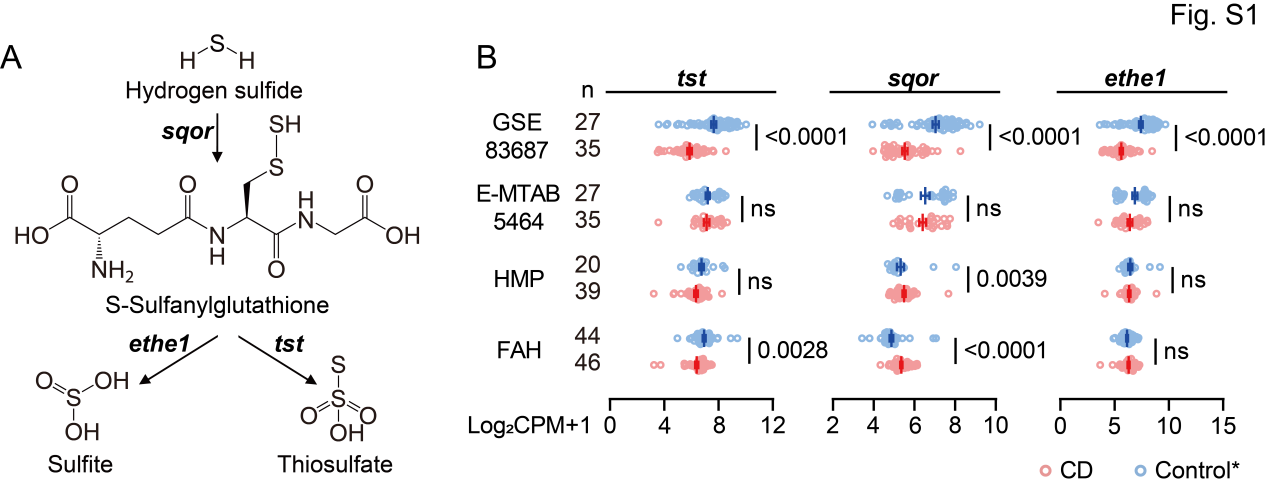
Fig. S1. CD is associated with changes in endogenous sulfide catabolism gene expression

(A) Endogenous hydrogen sulfide (H_2_S) catabolism in host.

(B) Analysis of tst, sqor, ethe1 and selenbp1 gene expression in CD and non-IBD control subjects’ mucosa in different IBD cohorts. *tst*, thiosulfate sulfurtransferase; *sqor*, thiosulfate sulfide:quinone oxidoreductase; *eteh1*, persulfide dioxygenase. * Refer to Fig. 2B for details regarding the control groups in each cohort.

Significance was determined by nonparametric Mann-Whitney test.


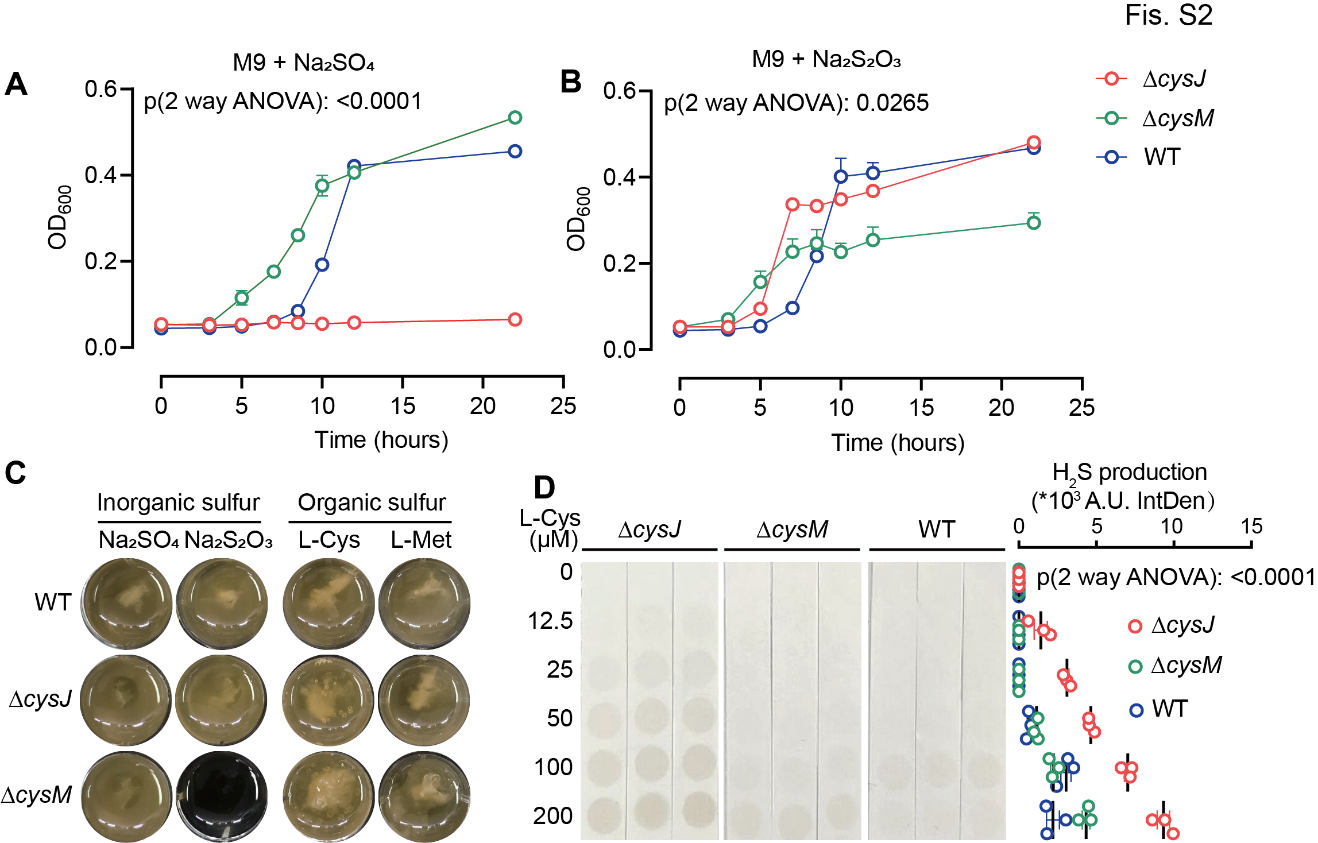


Fig. S2. Deletion of *cysJ* and *cysM* alters *E. coli* H_2_S sulfidogenic capacities

(A, B) Growth of *E. coli* WT and mutant strains on M9 medium with 1 mM sulfate (A) or sodium thiosulfate (B) as the sole sulfur source.

(C) WT and mutant strains of *E. coli* were qualitatively tested for H_2_S in SIM media of different sulfur sources under anaerobic conditions, as evidenced by the formation of black FeS.

(D) Relatively quantitative test of H_2_S produced by *E. coli* WT and mutant strains in M9 medium with different concentrations of L-cysteine as a sole sulfur source under anaerobic conditions.

Values are the means ± SEM from at least three independent experiments. Significance was measured with two-way ANOVA analysis.


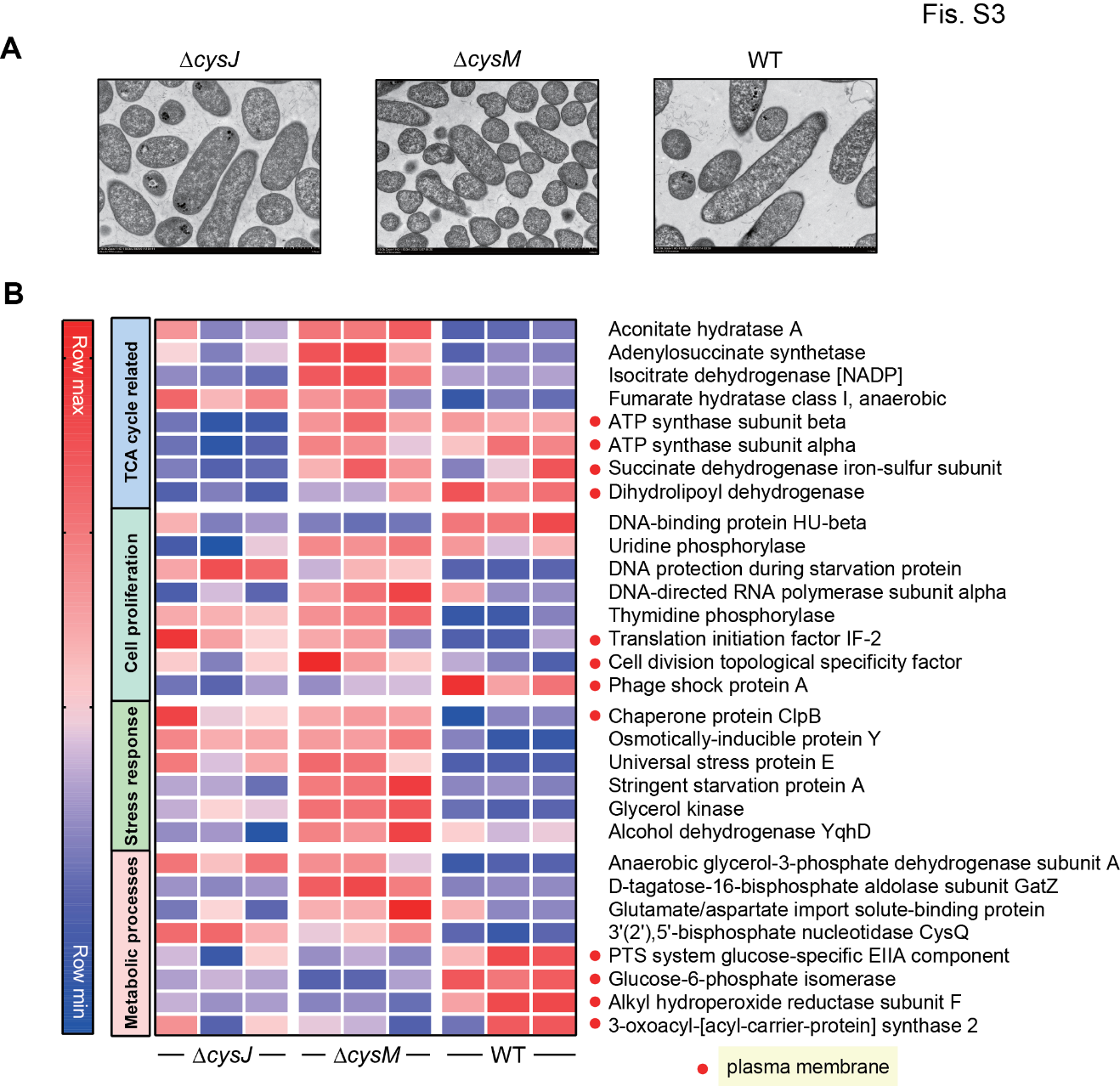


**Fig. S3. Deletion of *cysJ* and *cysM* alters *E. coli* morphological characteristics and proteomic profiles**

(A) Morphology of *E. coli* WT and mutant strains under transmission electron microscope.

(B) Heatmap of expression values (normalized abundances) of proteins among the mutants and wild-type strains. The chart shows coverage [%] >50 with good homogeneity across three replicates. The details are listed in supplementary dataset 5.


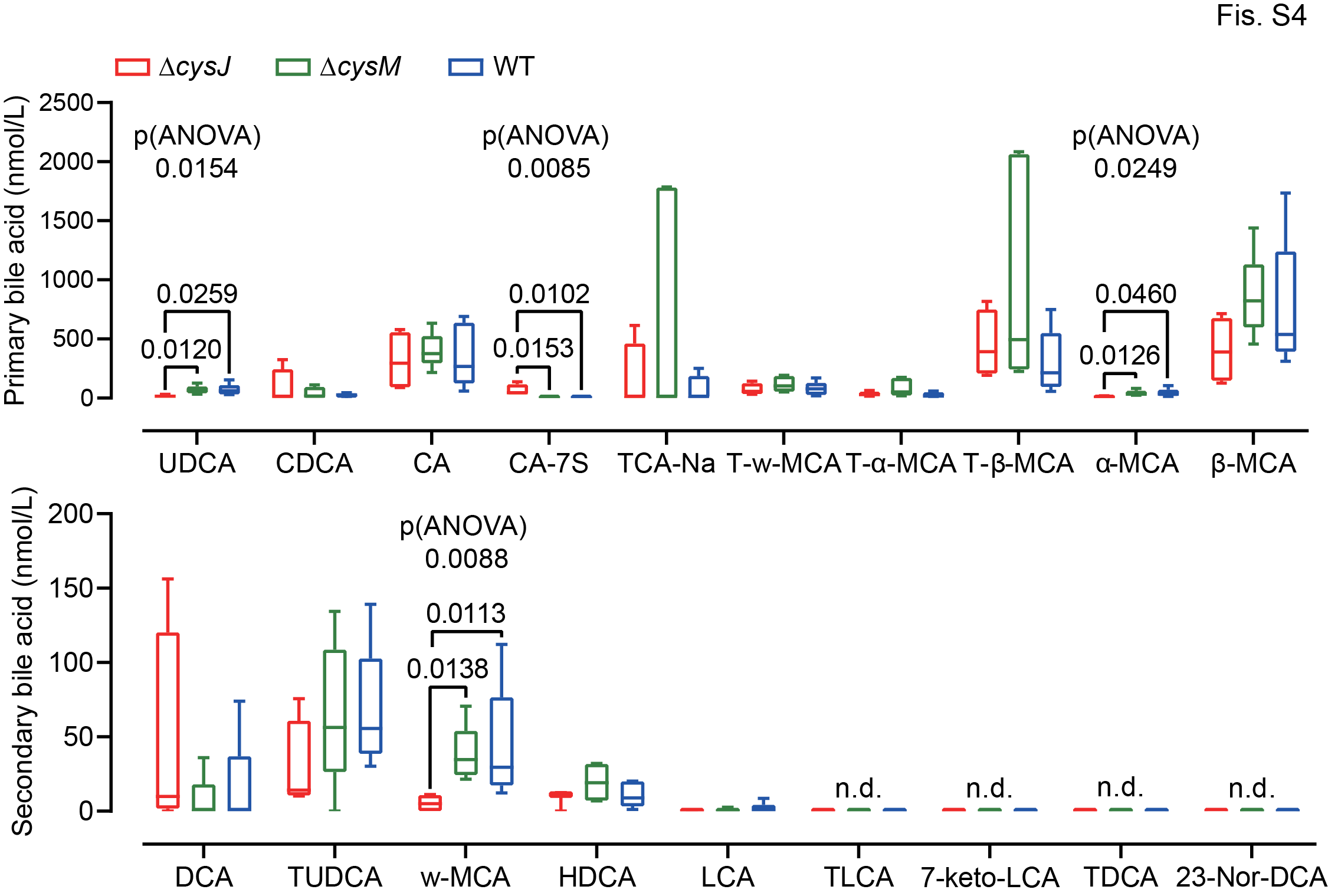


**Fig. S4. *E. coli* ASR-pathway modulates the serum level of bile acids (BAs) *in vivo***

The box-whiskers plot shows the serum bile acid concentration (ng/ml) in mice, n=5 (only 4 mice in *∆cysJ* group). Primary bile acid (above). Secondary bile acid (below). The maximum and minimum values in each set of data correspond to the top and bottom of the vertical line, respectively, and the middle horizontal line represents the median. Part of the non-detected bile acids are not listed, and the details of the standards are listed in supplementary dataset 7. P-value was determined by ordinary one-way ANOVA analysis with Tukey's multiple comparisons.

Dataset S1. Gene names and their corresponding KEGG IDs for both bacterial and host sulfidogenic genes.

Supplementary dataset 1

Dataset S2. Correlation analysis of microbial sulfidogenic genes within identical gene clusters in the metagenomic data of the FAH-SYSU and PRISM cohorts.

Supplementary dataset 2

Dataset S3. Quantification of microbial sulfidogenic genes in the metagenomic data of the FAH-SYSU and PRISM cohorts.

Supplementary dataset 3

Dataset S4. Phylogenetic distribution of genomes harboing asr- and dsr-associated genes in 1635 reference genomes of the Human Microbiome Project (HMP).

Supplementary dataset 4

Dataset S5. Proteomic profiles of *E. coli* wild type, as well as *∆cysJ* and *∆cysM* mutants.

Supplementary dataset 5

Dataset S6. Compilation of identified bacterial sulfidogenic genes used as query sequences for developing markers for metagenomic profiling.

Supplementary dataset 6

Dataset S7. MRM transitions for standard bile acids and internal standards.

Supplementary dataset 7

**SI References**

1. Nemet, I., Saha, P.P., Gupta, N., Zhu, W., Romano, K.A., Skye, S.M.*, et al.* A Cardiovascular Disease-Linked Gut Microbial Metabolite Acts via Adrenergic Receptors. *Cell.* 2020;180: 862-877 e822.

2. Wirtz, S., Popp, V., Kindermann, M., Gerlach, K., Weigmann, B., Fichtner-Feigl, S.*, et al.* Chemically induced mouse models of acute and chronic intestinal inflammation. *Nat Protoc.* 2017;12: 1295-1309.

3. Reid, P.E., Owen, D.A., Fletcher, K., Rowan, R.E., Reimer, C.L., Rouse, G.J.*, et al.* The histochemical specificity of high iron diamine-alcian blue. *Histochem J.* 1989;21: 501-507.

4. Xu, P., Xi, Y., Zhu, J., Zhang, M., Luka, Z., Stolz, D.B.*, et al.* Intestinal Sulfation Is Essential to Protect Against Colitis and Colonic Carcinogenesis. *Gastroenterology.* 2021;161: 271-286 e211.
